# The Genus *Cladosporium*: A Rich Source of Diverse and Bioactive Natural Compounds

**DOI:** 10.3390/molecules26133959

**Published:** 2021-06-28

**Authors:** Maria Michela Salvatore, Anna Andolfi, Rosario Nicoletti

**Affiliations:** 1 Department of Chemical Sciences, University of Naples ‘Federico II’, 80126 Naples, Italy; mariamichela.salvatore@unina.it; 2 BAT Center—Interuniversity Center for Studies on Bioinspired Agro-Environmental Technology, University of Naples ‘Federico II’, Portici, 80055 Naples, Italy; 3 Council for Agricultural Research and Economics, Research Centre for Olive, Fruit and Citrus Crops, 81100 Caserta, Italy; rosario.nicoletti@crea.gov.it; 4 Department of Agricultural Sciences, University of Naples ‘Federico II’, 80055 Portici, Italy

**Keywords:** natural products, bioactive secondary metabolites, fungal species, fungal extrolites

## Abstract

Fungi are renowned as one of the most fruitful sources of chemodiversity and for their ubiquitous occurrence. Among the many taxonomic groupings considered for the implications deriving from their biosynthetic aptitudes, the genus *Cladosporium* stands out as one of the most common in indoor environments. A better understanding of the impact of these fungi on human health and activities is clearly based on the improvement of our knowledge of the structural aspects and biological properties of their secondary metabolites, which are reviewed in the present paper.

## 1. Introduction

Results of recent research in the mycological field have further disclosed the pervasive diffusion of fungi in the genus *Cladosporium* (Dothideomycetes, Cladosporiaceae). Basically saprophytic, these Ascomycetes are spread in every kind of terrestrial and marine environment, where they establish various symbiotic relationships with plants and animals [1]; moreover, they are among the most frequent fungi detected in indoor spaces [2,3]. This latter connotation implies obvious opportunities for interactions with people, which can sometimes evolve into undesirable effects in terms of allergic or even pathogenic reactions [4,5,6,7,8].

Over the past two decades, investigations into the occurrence of *Cladosporium* spp. have been boosted by their tremendous ecological adaptability, as well as their frequent implication in human activities and medical aspects. Fundamental support from the molecular tools for species identification has enabled mycologists to disclose an exceptional taxonomic variation, with as many as 218 accepted species considered in the most recent update [3] and more new species added to the list in the last three years [9,10,11]. Considering the importance of secondary metabolites as mediators of biological interactions, this versatility has also generated notable research activity concerning the metabolome of these fungi and its biological properties, which are revised in the present paper.

## 2. Fifty Years of Metabolomic Studies in *Cladosporium*

**Figure 1:**
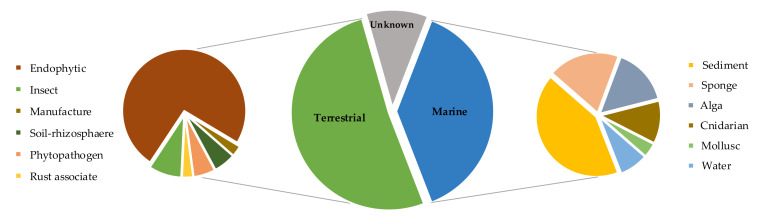
Pie charts of the origin of the strains examined in the present review.

A set of 68 *Cladosporium* strains have been examined so far, about 2/3 of which have been formally classified at the species level and ascribed to 12 taxa (Table 1). In this respect, the most frequent species are represented by the progenitors of the three main species complexes of the genus [1,3]. This may imply that in some cases the taxonomic identification has been approximate, as it only relied on morphological characters or ITS sequences. Concerning the origin, the examined strains are almost equally distributed between terrestrial and marine sources, with a prevalence of those recovered as endophytes or from sediments (Figure 1).

Despite the low number of strains, a long list of products has been reported from *Cladosporium*, starting with the finding of cladosporin in 1971 [12]. In fact, from analysis of the available literature, a total of 244 chemically defined compounds can be extracted, belonging to different classes of secondary metabolites, such as azaphilones, benzofluoranthenones, coumarins and isocumarins, lactones, naphthalenones, macrolides, perylenequinones, sterols and others (Table 2). Of course, this list includes both known metabolites and compounds, which have been first characterized from these fungi, with the latter representing a remarkable share (147, corresponding to about 60%). In our survey, we avoided considering some products that are known intermediates in biosynthetic processes, clearly represent possible contaminants of the fungal cultures, or were just tentatively identified [13,14,15,16,17,18,19,20,21].

Some errors and overlapping in the compound names attribution have arisen during the accurate examination of the available literature on this topic. In particular, we can consider two recurring issues, which are “more names, one chemical structure” and “one name, more chemical structures”. For instance, cladosporin certainly belongs to the first case because its chemical structure is also known by the name asperentin [97]. For this reason, in Table 2, we added in brackets eventual additional names for compounds that fall under this case.

On the other hand, due to the intense research activity concerning this fungal genus, it has happened that some authors conducted their research parallel to the finding of closely related compounds. The temporal proximity in publishing has sometimes caused the attribution of the same name to different chemical structures (e.g., cladosporiumin I). In the case of cladosporol G, this issue was rather a consequence of author inaccuracy, since the elapsed time of about one year between the consecutive reports would have afforded an accurate preliminary check. In all cases of homonymy in Table 2, we have added the Latin suffix “*bis*” to the compounds that have been characterized later, as inferred from the date of submission to the journal.

Additional nomenclatural issues are represented by the absence of a proposed name, or authors’ choice to follow IUPAC rules instead of introducing trivial names derived from closely related compounds. Indeed, the use of trivial names represents a very common and useful guideline in natural product research because systematic names can be so convoluted and difficult to parse.

### 2.1. Alkaloids

Aspernigrin A (**1**) was originally characterized from the culture of a sponge-derived *Aspergillus niger* strain with its structure assigned mainly from its NMR and MS data [100], but it was structurally revised after reisolating from an endophytic strain of *Cladosporium herbarum* [47].

**Figure 2:**
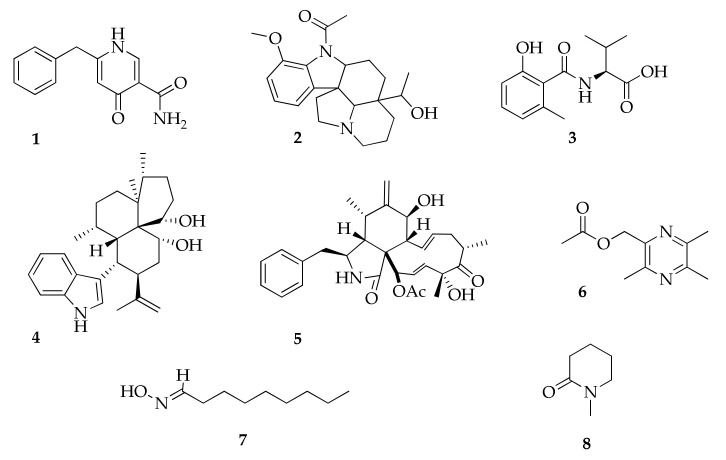
Structures of alkaloids.

Cladosporine A (**4**) is the first indole diterpenoid alkaloid reported as a product of a *Cladosporium* strain [84]. In the class of alkaloids (Figure 2), cladosin E (**3**) and 2-methylacetate-3,5,6-trimethylpyrazine (**6**) are other new natural products from strains of *Cladosporium sphaerospermum* [60] and an endophytic *Cladosporium* sp. [85].

### 2.2. Azaphilones

**Figure 3:**
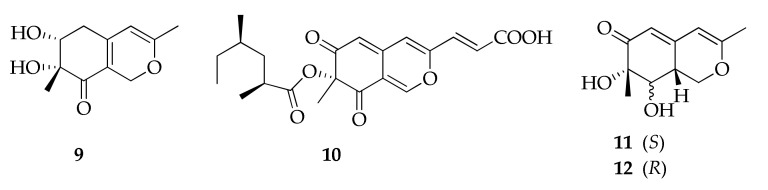
Structures of azaphilones.

Azaphilones are a structurally variable family of fungal polyketide metabolites possessing a highly oxygentated pyranoquinone bicyclic core and a quaternary carbon center. Well-known from genera such as *Aspergillus*, *Penicillium* and *Talaromyces* [101,102], these compounds have also been reported from the *Cladosporium* species (Figure 3) [49,52]. In particular, two new azaphilones, named perangustols A and B (**11**,**12**), were isolated from a marine-derived isolate of *Cladosporium perangustum* together with the new natural product, bicyclic diol (**9**) [52].

### 2.3. Benzofluorantheneones

**Figure 4:**
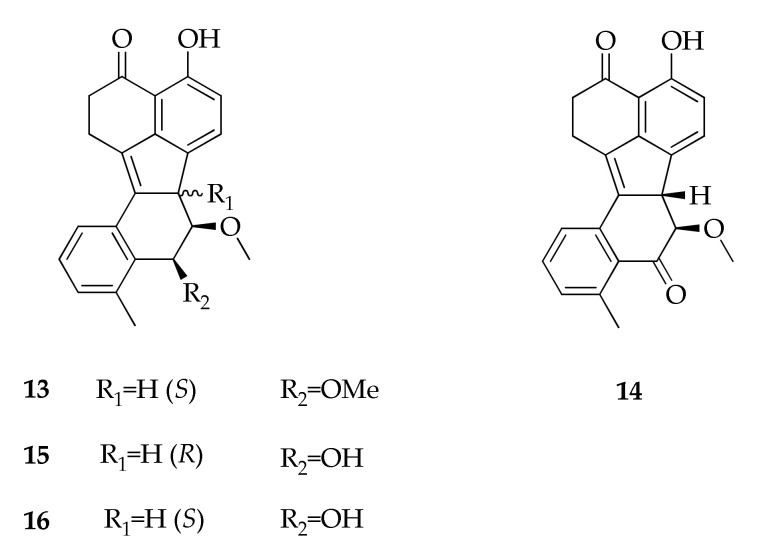
Structures of benzofluoranthenones.

During a screening of microbial extracts, a series of novel reduced benzofluorantheneones (**13**–**16**) was identified in the fermentation broth of a strain of *Cladosporium cladosporioides* recovered from a dead insect (Figure 4) [27,28].

### 2.4. Benzopyrones

**Figure 5:**
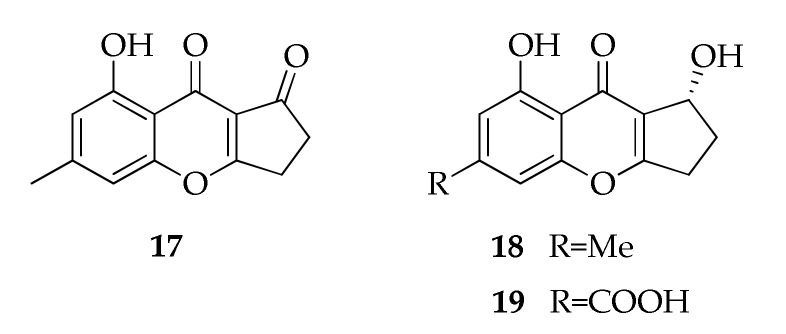
Structures of benozopyranones.

A member of the benzopyrenes family named coniochaetone K (**19**) was isolated for the first time as a product of a coral symbiotic strain of *Cladosporium halotolerans* (Figure 5) [43]. This compound is particularly interesting because it has an uncommon carboxylic group in the backbone at position C-8’. It was identified together with the already known coniochaetones A-B (**17**,**18**) and several compounds belonging to the xanthones group. However, it must be underlined that a compound with the same name was previously characterized from a strain of *Penicillium oxalicum*, which differs in the absence of a carboxylic group and the presence of an additional hydroxyl group in the cyclopentane ring [103].

### 2.5. Binaphtopyrones

**Figure 6:**
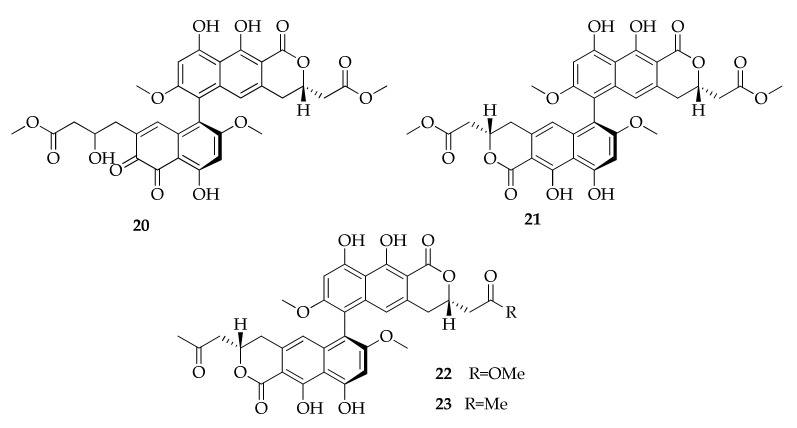
Structures of binaphthopyrones.

So far, members of the family of binaphthopyrones (Figure 6) were isolated only from an extremophilic strain of *C. cladosporioides* collected from a hypersaline lake in Egypt. In particular, cladosporinone (**20**), together with some viriditoxin derivatives (**21**–**23**), was isolated for the first time from this strain grown in a fermentation broth fortified with 3.5% sea salt [22]. The finding of compounds with original structures from fungi in extreme habitats is not unusual, considering that these microorganisms require special survival strategies for growing and reproducing, and adaptation to such conditions requires the modification of gene regulation and metabolic pathways [104].

### 2.6. Butanolides and Butenolides

**Figure 7:**
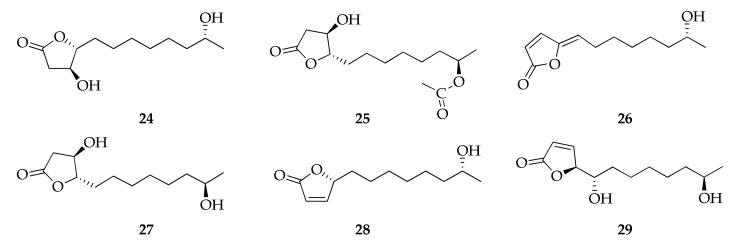
Structures of butanolides and butenolides.

Some metabolites from the cladospolide series are members of the family of butanolides and butenolides (Figure 7), a subgroup of lactones with a four-carbon ring structure. Many of them were isolated from several species of *Cladosporium* along with other cladospolides that are members of the series of macrolides [32,44,67,71,94].

### 2.7. Cinnamic acid Derivatives

**Figure 8:**
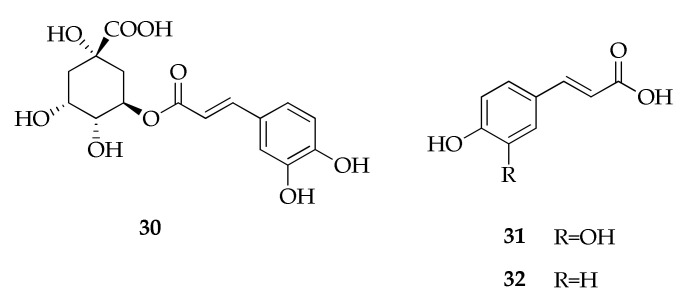
Structures of cinnamic acid derivatives.

Phenylalanine and tyrosine are precursors for a wide range of natural products. Commonly in plants and fungi, a frequent step is the elimination of ammonia from the side chain to generate cinnamic acids and related compounds. Caffeic and coumaric acids are among the most common naturally occurring cinnamic acids, which can also be found in a range of esterified forms, such as quinic acid forming chlorogenic acid [105]. Caffeic, chlorogenic and coumaric acids (**30**–**32**, Figure 8) were detected in the culture extract of an endophytic strain of *Cladosporium velox* isolated from stem of *Tinospora cordifolia*. Comparative analysis of the metabolite profiles of this strain showed similar composition with stem and leaf extracts of the host plant [70].

### 2.8. Citrinin Derivatives

**Figure 9:**
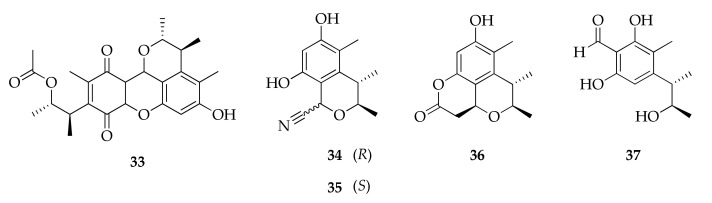
Structures of citrinin derivatives.

Four new compounds from a marine-derived strain of *Cladosporium* sp. were reported as citrinin derivatives (**34**–**37**, Figure 9) [92]. Citrinin is a polyketide mycotoxin first isolated from *Penicillium citrinum* [106]. Considering the existence of the name cladosporin for the product (**39**) since 1971 [12] and cladosporine A (**4**) [84], the use of the same name for this new series is questionable. Furthermore, a known citrinin dimeric derivative named citrinin H1 (**33**) was isolated from a strain of *Cladosporium* sp. [85].

### 2.9. Coumarins and Isocoumarins

**Figure 10:**
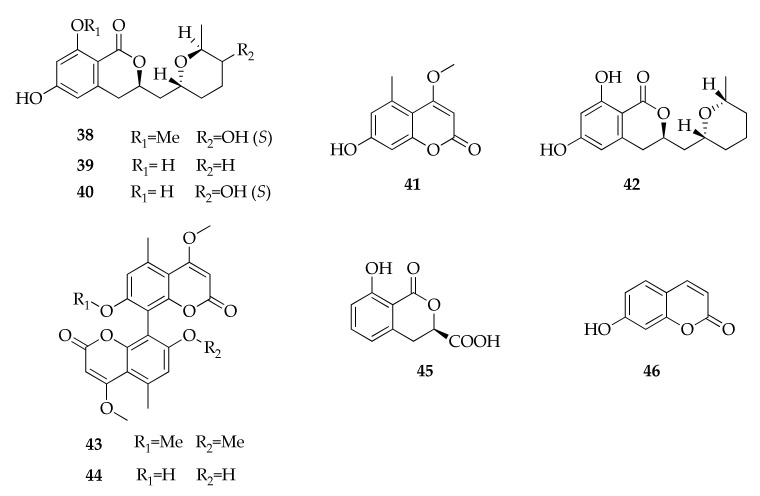
Structures of coumarins and isocoumarins.

Cladosporin (**39**) is a member of 3,4-dihydroisocoumarins, a subgroup of isocoumarins that are commonly produced by fungi, along with coumarins [107]. Coumarins and isocoumarins are structural isomers, and their general moieties are respectively characterized by a chromen-2-one and 1*H*-isochromen-1-one [108]. Cladosporin was reported for the first time from mycelium of *C. cladosporioides* [12], but its absolute stereochemistry was elucidated only 17 years later using ^2^H decoupled ^2^H, ^13^C NMR shift correlation [36]. Cladosporin has also been isolated from the culture filtrate of another strain of *C. cladosporioides* together with its epimer in C-6′ named isocladosporin (**42**, Figure 10) [24,37]. It must be noted that **39** was later found from *Aspergillus flavus* [97] and an unidentified *Aspergillus* strain [109], but it was wrongly reported as a new compound with the name asperentin. As a consequence, some of its analogues were characterized as asperentin-8-methyl ether (**38**) and 5′-hydroxyasperentin (**40**) [25].

Kotanin (**43**) and orlandin (**44**) are two closely related dimeric coumarins produced by an endophytic strain of *C. herbarum* isolated from the leaves of *Cynodon dactylon* [47], which were previously reported as products of plant-associated *Aspergillus* strains [110,111].

### 2.10. Cyclohexene Derivatives

**Figure 11:**
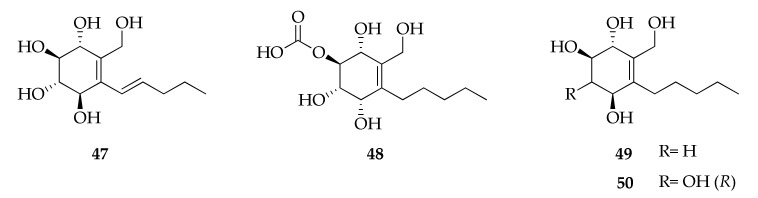
Structures of cyclohexene derivatives.

Four new cyclohexene derivatives named cladoscyclitols A–D (**47**–**50**) were obtained from the culture broth of a mangrove endophytic fungus *Cladosporium* sp. (Figure 11) [82].

### 2.11. Depsides

**Figure 12:**
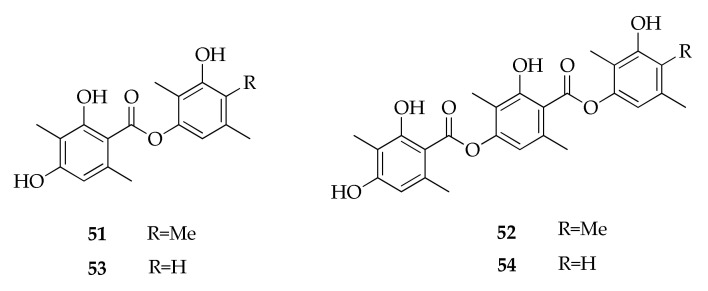
Structures of depsides.

Four new despsides (**51**–**54**) were isolated from an endophytic strain of *Cladosporium uredinicola* (Figure 12) [69]. The authors later revised the structures of 3-hydroxy-2,4,5-trimethylphenyl 4-[(2,4-dihydroxy-3,6-dimethylbenzoyl)oxy]-2-hydroxy-3,6-dimethylbenzoate (**52**) and 3-hydroxy-2,5-dimethylphenyl 4-[(2,4-dihydroxy-3,6-dimethylbenzoyl)oxy]-2-hydroxy-3,6-dimethylbenzoate (**54**) [98].

### 2.12. Diketopiperazines

**Figure 13:**
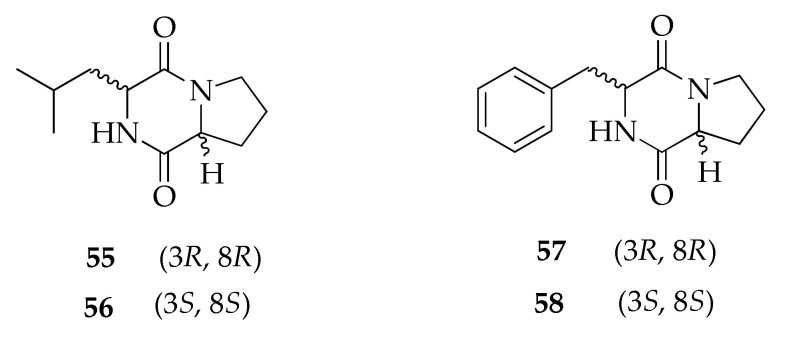
Structures of diketopiperazines.

The diketopiperazines (**55**–**58**) reported in Figure 13 were identified via GC-MS in the crude extract of the culture filtrate of a strain of *C. cladosporioides* along with several volatile metabolites [17]. The structure of compounds in this class is based on a cyclic scaffold deriving from the condensation of two α-amino acids.

### 2.13. Flavonoids

**Figure 14:**
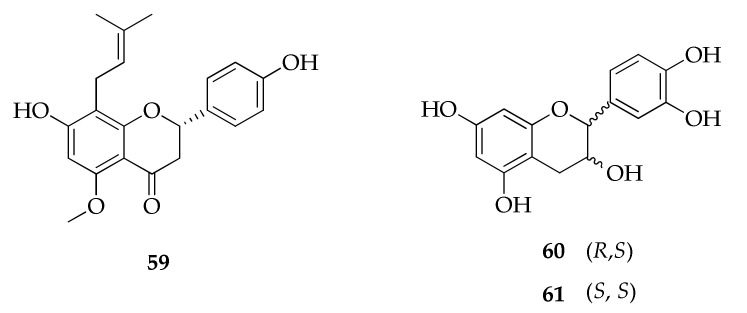
Structures of flavonoids.

The investigation of compounds produced by a previously mentioned endophytic strain of *C. velox* isolated from *Tinospora cordifolia* led to the identification, via RP-HPLC, of the known flavonenes called catechin (**60**) and epicatechin (**61**) by comparison of their retention times with those of commercially available standard compounds (Figure 14) [70].

The known (2*S*)-7,4′-dihydroxy-5-methoxy-8-(γ,γ-dimethylallyl)-flavanone (**59**) is a prenylated flavanone in which prenylation is represented by 3,3-dimethylallyl substituent at position 8’ [93].

### 2.14. Gibberellins

**Figure 15:**
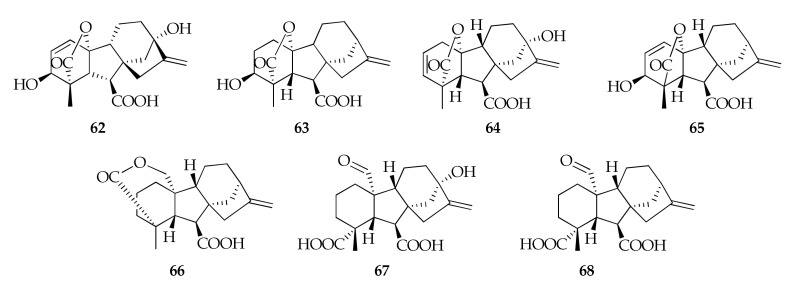
Structures of gibberellins.

A strain of *C. sphaerospermum* from salt-stressed soybean plants was able to induce maximum plant growth in both soybean and Waito-C rice. Interestingly, high amounts of gibberellins (**62–68**) were detected in its culture filtrate (Figure 15) [57]. Gibberellins are diterpenoid hormones involved in many aspects of plant growth and development, hence playing a role in the mutualistic plant-endophyte interactions [112].

### 2.15. Fusicoccane Diterpene Glycosides

**Figure 16:**
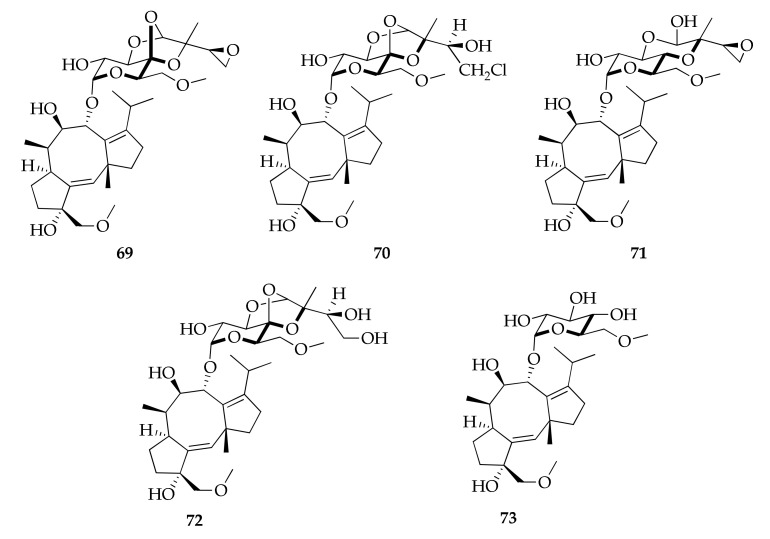
Structures of fusicoccane diterpene glycosides.

Cotylenin A (**69**) is the major and most structurally complex metabolite of fusicoccane diterpene glycosides isolated from the *Cladosporium* species (Figure 16). Cotylenins A–D (**69**–**72**) are characterized by the presence of a common aglycone named cotylenol and an unusual sugar moiety consisting in a 6-*O*-methyl-α-d-glucosyl derivative with an oxygenated C5-isoprene unit [72,73,74].

### 2.16. Lactones

**Figure 17:**
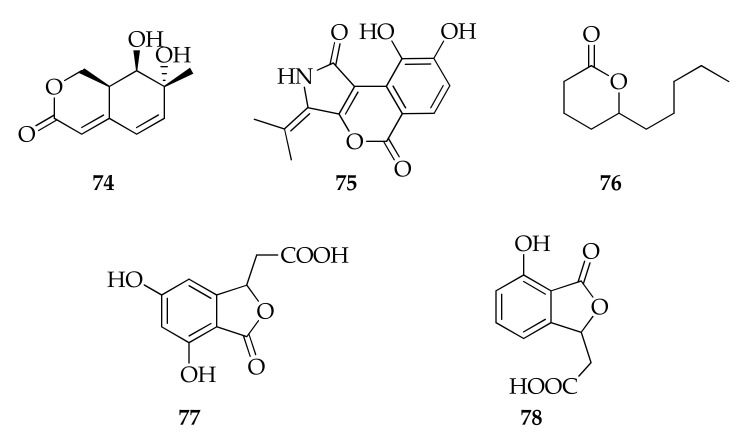
Structures of lactones.

This class includes structurally diverse compounds with a 1-oxacycloalkan-2-one structure in common. Cladosporactone A (**74**), cladosporimide A (**75**) and herbaric acid (**76**) are lactones isolated for the first time from a marine-derived strain of *Cladosporium* sp. (Figure 17) [35,45,93].

### 2.17. Macrolides

Macrolides are a large family of compounds characterized by a macrocyclic lactone ring. Rings are commonly 12, 14, or 16 membered [105].

**Figure 18:**
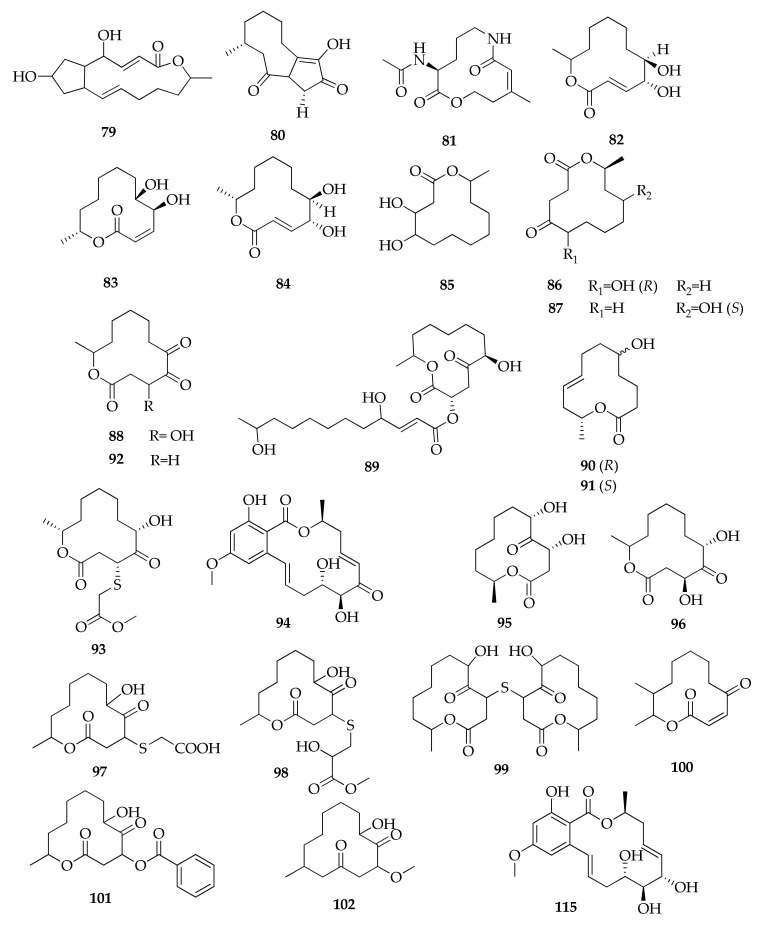
Structures of macrodiolides.

**Figure 19:**
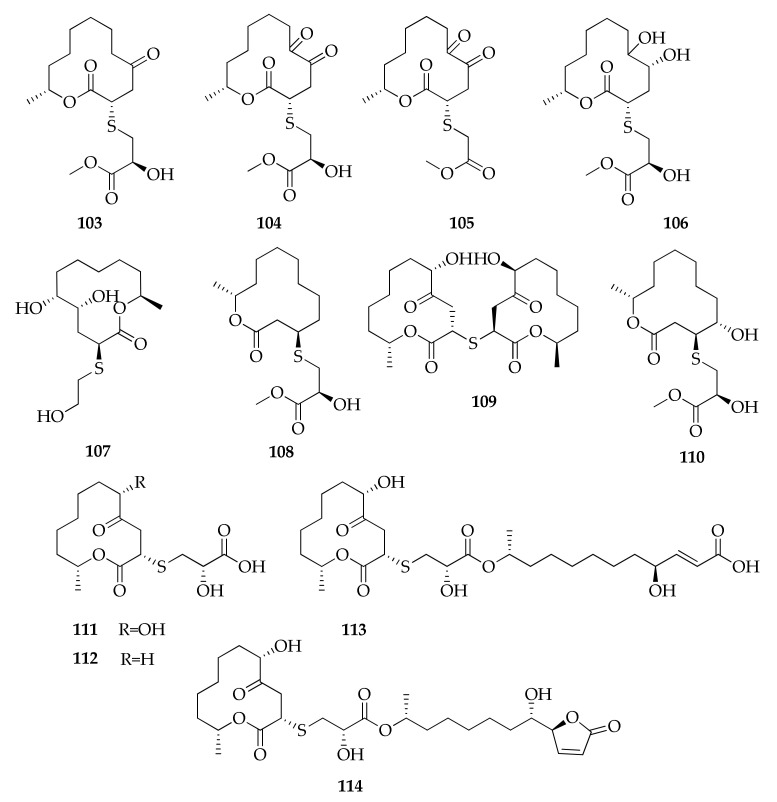
Structures of thiocladospolides.

Macrolides with a different number of members were also isolated from cultures of *Cladosporium* spp. (Figure 18 and Figure 19), many of them reported for the first time. In fact, several 12-membered macrolides were reported from marine-derived strains of the *Cladosporium* species, such as recifeiolide analogues, namely 5*R* and 5*S*-hydroxyrecifeiolides (**90**–**91**) [32] and sporiolides A and B (**101**–**102**) [88]. 

The list of macrolides from the *Cladosporium* species includes pandangolide 1a and pandangolides 1–4 (**95**–**99**). Pandangolide 1 and 2 were already known as products of an unidentified fungal species obtained from a marine sponge [113], while pandangolides 3 and 4 were identified for the first time from *C. herbarum* [44]. Pandangolide 1a was isolated, together with its known diastereomer **95**, from a sponge-associated *Cladosporium* sp. [71].

The investigation of metabolites produced by the mangrove endophytic *Cladosporium* sp. led to the isolation of new compounds called thiocladospolides A–E (**103**–**107**, Figure 19) and the macrodiolide lactam derived from ornithine, called cladospamide A (**81**), together with the known cladospolide B (**83**) [33,91]. This latter compound was previously isolated and identified during a screening for new plant growth regulators produced by *C. cladosporioides*, along with its isomer cladospolide A (**82**) [114,115,116]. The cladospolide series also includes the diastereomer of **82**, named cladospolide C (**84**), which was isolated from *Cladosporium tenuissimum* [64].

Two new macrolides (i.e., 4-hydroxy-12-methyloxacyclododecane-2,5,6-trione (**88**) and 12-methyloxacyclododecane-2,5,6-trione (**92**)), were isolated from an endophytic strain of *C. colocasiae*, together with known compounds identified as cladospolide A (**82**), (6*R*,12*S*)-6-hydroxy-12-methyl-1-oxacyclododecane-2,5-dione (**86**), pandangolide 1 (**95**), patulolide B (**100**) and seco-patulolide C (162) [41].

An unusual macrolide with a bicyclo 5–9 ring system, named cladocladosin A (**80**), was isolated from the mangrove-derived endophytic fungus *C. cladosporioides*, along with two new sulfur-containing macrodiolides, namely thiocladospolides F and G (**108**,**110**) [34]. Moreover, five new thiocladospolides were identified together with some known compounds from a strain of *Cladosporium oxysporum* (Figure 19) [50]. These new compounds were named thiocladospolides F-J, even if thiocladospolides F and G (**109**,**111**) had been previously reported with different structures, representing another example of the issue “one name, more structures”. For this reason, these compounds are reported in Table 2 as thiocladospolides F *bis* and G *bis*.

### 2.18. Naphthalene Derivatives

**Figure 20:**
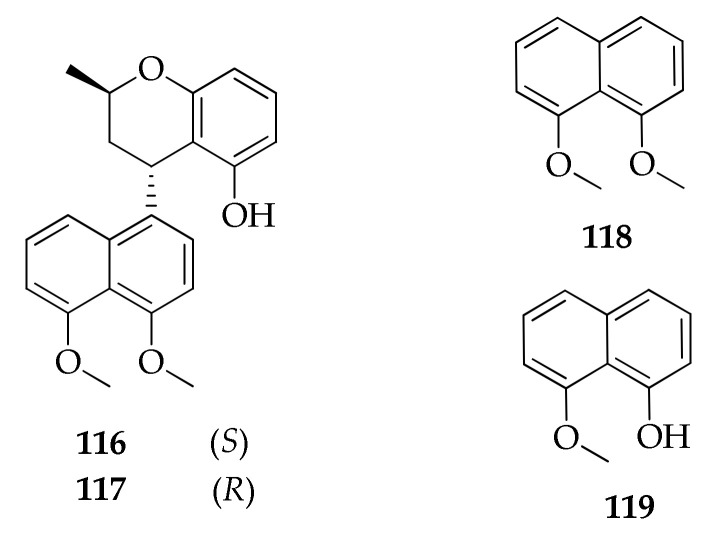
Structures of naphthalene derivatives.

Two new dimeric naphthalene derivatives, named cladonaphchroms A and B (**116**, **117**), were obtained from a mangrove-derived strain of *Cladosporium* sp. (Figure 20). These compounds were detected in the fungal culture extract along with some known metabolites, such as 5-hydroxy-2-methyl-4*H*-chromen-4-one (**236**), (*R*)-5-hydroxy-2-methyl-chroman-4-one (**237**), 1,8-dimethoxynaphthalene (**118**) and 8-methoxynaphthalen-1-ol (**119**) [83]. Additionally, **118** was also obtained as product of a mangrove-derived strain of *Cladosporium* sp. [81].

### 2.19. Naphthalenones

**Figure 21:**
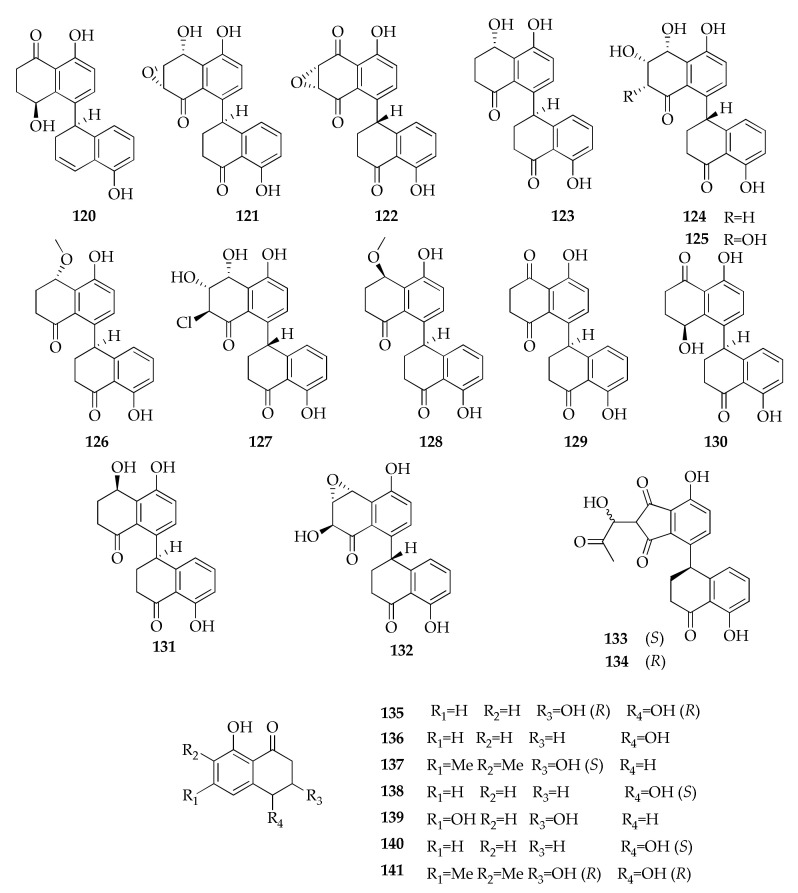
Structures of naphthalenones.

(−)-*trans*-(3*R*,4*R*)-3,4,8-Trihydroxy-6,7-dimethyl-3,4-dihydronaphthalen-1(2*H*)-one (**141**) is a new compound isolated from a mangrove-derived *Cladosporium* sp. (Figure 21), produced along with six known compounds (i.e., **118**,**135**,**137**,**140**) [81]. Scytalone (**139**) is another compound from this class, isolated from an endophytic strain of *C. tenuissimum* from *Pinus wallichiana* [68]. It is a polyketide known as an intermediate in melanin biosynthesis produced by many fungi associated with plants [117,118].

Dimeric tetralones are a subclass of naphthalenones made from two monomers of bicyclic aromatic hydrocarbon and a ketone. A marine-derived strain of *Cladosporium* sp. also produces new dimeric tetralones: the newly isolated altertoxin XII (**120**) and the known cladosporol I (**130**) [87].

Among the compounds in this family, cladosporol A (**121**) was isolated for the first time from *C. cladosporioides* [26] and later on from *C. tenuissimum* together with some analogues, cladosporol B–E (**122**–**125**) [66]. Their absolute configurations were revised some years later from (4’*R*) to (4’*S*) when five new dimeric tetralones (i.e., cladosporols F–J) and the known cladosporol C (**123**) were isolated from an algal endophytic strain of *C. cladosporioides* [39]. Four new dimeric tetralones, namely clindanones A and B (**133**,**134**) and cladosporols F and G (**126**,**127**), were identified by a deep-sea derived strain of *C. cladosporioides* along with the known isosclerone (**138**), which is the only monomeric tetralone isolated from the *Cladosporium* species so far [40]. Clindanones (**133**,**134**) possess new dimeric forms of the skeleton composed by the coupling of indanone and 1-tetralone units. As introduced in chapter 2, cladosporol G (**128**) produced by the algal strain [39] is different from the compound (**127**) with the same name previously discovered as a product of the deep-sea derived strain.

Some cladosporols (i.e., **121**, **123** and **124**) were also isolated from a strain of *Cladosporium* sp. derived from the mangrove plant *Kandelia candel*, along with the new dimeric tetralone named cladosporone A (**132**) [86].

### 2.20. Naphtoquinones and Anthraquinones

**Figure 22:**
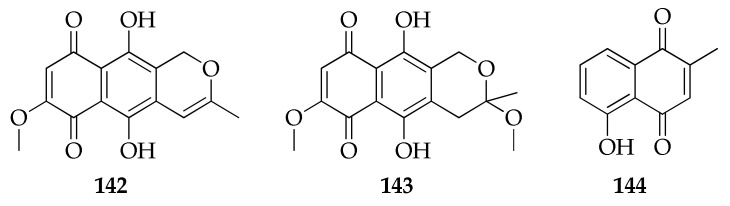
Structures of naphthoquinones and anthraquinones.

Naphthoquinones and anthraquinones have been widely identified as metabolites from various plants, microbes and marine organisms [102,119,120]. Two anthraquinones, namely anhydrofusarubin (**142**) and methyl ether of fusarubin (**143**), were isolated from *Cladosporium* sp. from the bark of the plant *Rauwolfia serpentina* [90]. The only naphthoquinone known from *Cladosporium*, plumbagin (**144**), was isolated from a strain of *Cladosporium delicatulum*, which resulted as the most potent producer of this valuable drug after a dedicated screening of endophytic fungi carried out to find strains able to synthesize this valuable drug (Figure 22) [42].

### 2.21. Perylenquinones

**Figure 23:**
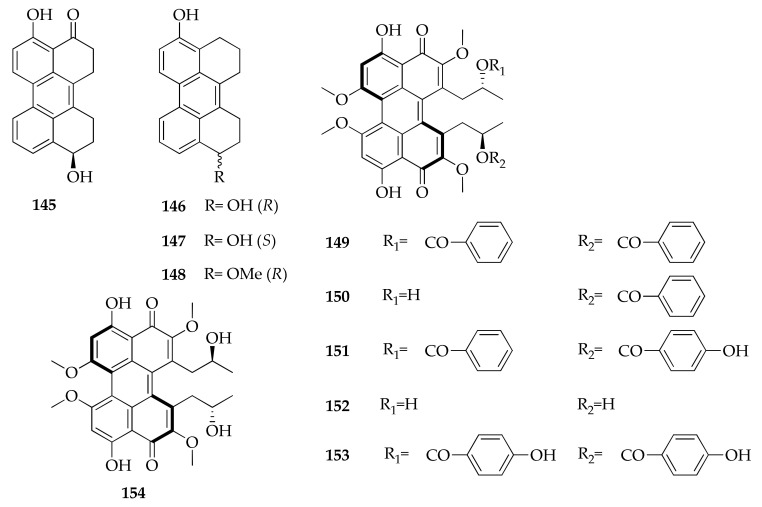
Structures of perylenequinones.

The first member of the family of perylenquinones (Figure 23), named phleichrome (**154**), was reported as a new phytotoxic compound produced by *Cladosporium phlei* [53,99]. The stereochemistry of phleichrome was investigated in detail in a subsequent study, which reports the conversion of **154** in isophleichrome, highlighting the similarity in behavior and physical data with another couple of fungal perylenquinones, cercosporin and isocercosporin [54]. In fact, perylenquinones show intriguing stereochemical features, such as axial chirality due to the helical shape of the constrained pentacyclic ring, combined with asymmetric carbons in the side chains. Even if it was indicated that phleichrome can be thermally converted in its unnatural diastereoisomer named isophleichrome [54], the production by *C. cladosporioides* of ent-isophleichrome (**152**) was reported [46]. Moreover, several esters of **152**, belonging to the series of calphostins, were isolated from a strain of *Cladosporium* sp. Calphostin C and I (**151**,**153**) [30,121] have also been incorrectly reported as new products with the names cladochromes E and D [31]. In fact, these compounds had been previously isolated, and their physico-chemical properties investigated in the course of screening the potential inhibitors of protein kinase C (PKC) from a strain of *C. cladosporioides*, along with several other calphostins (**149**–**153**) [29,30]. Moreover, four new perylenquinones, altertoxins VIII-XI (**145**–**148**), were isolated from the fermentation broth of a marine-derived strain of *Cladosporium* sp. [87]. These new metabolites partially share structures with a series of metabolites originally isolated from the *Alternaria* species [122].

### 2.22. Pyrones

**Figure 24:**
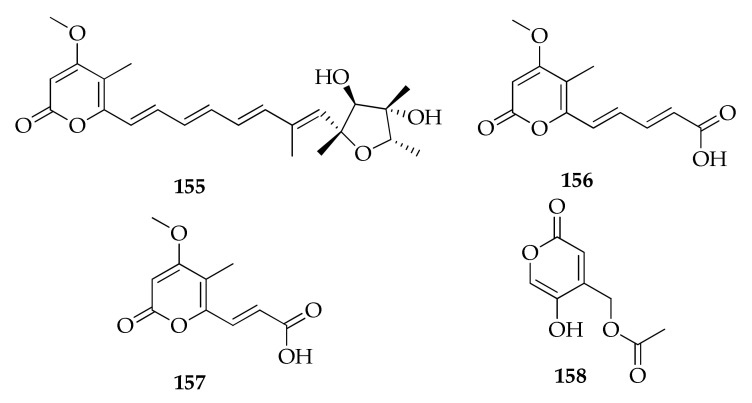
Structures of pyrones.

Pyrones represent a family of six-membered unsaturated cyclic compounds containing oxygen that naturally occurs in two isomeric forms, either as 2-pyrone or 4-pyrone. 2-pyrone is extremely prevalent in numerous natural products isolated from plants, animals, marine organisms, bacteria, fungi, and insects [123,124]. Two new 2-pyrones (i.e., herbarins A and B (**156**,**157**)) were obtained from a spongiculous strain of *C. herbarum* isolated (Figure 24) [45].

### 2.23. Seco Acids

**Figure 25:**
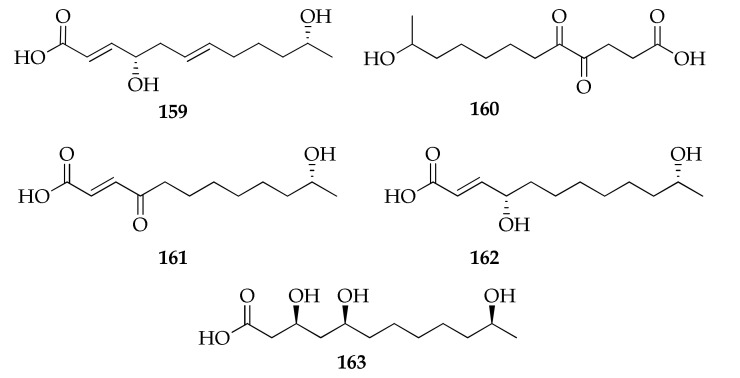
Structures of seco acids.

The 12 membered seco acids reported in Figure 25 were found to be produced by strains of *C. cladosporioides* and *C. tenuissimum*, along with members of the families of lactones or macrolides [33,67,68,94]. It can be speculated that these compounds are intermediates in the biosynthesis of cyclic compounds because seco acids are the starting material for the production of lactones [125].

### 2.24. Sterols

**Figure 26:**
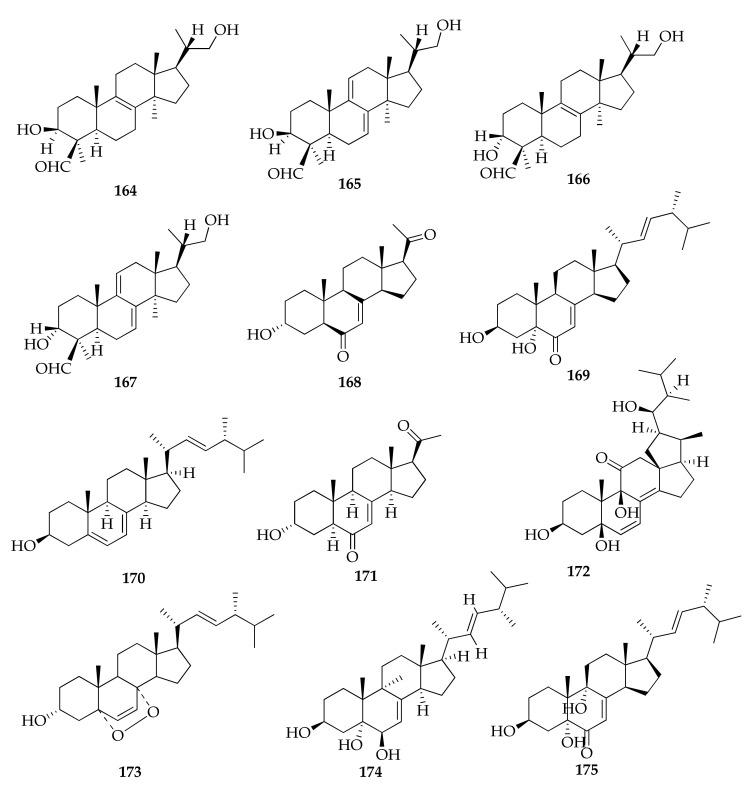
Structures of sterols.

Sterols are a class of lipids involved in several metabolic reactions since they are components of the membrane of eukaryotic organisms playing a crucial role in permeability and fluidity [126]. They are modified triterpenoids containing the tetracyclic ring system of lanosterol but lacking the three methyl groups at C-4 and C14. The predominant sterol found in fungi is ergosterol, which has frequently been investigated in human pathogenic fungal strains [127]. Ergosterol (**170**) was also identified as product of a strain of *Cladosporium* sp., along with 23,24,25,26,27-pentanorlanost-8-ene-3β,22-diol (**172**), peroxyergosterol (**173**) and four new pentanorlanostane derivatives named cladosporides A–D (**164**–**167**) (Figure 26) [79,80].

### 2.25. Tetramic Acids

**Figure 27:**
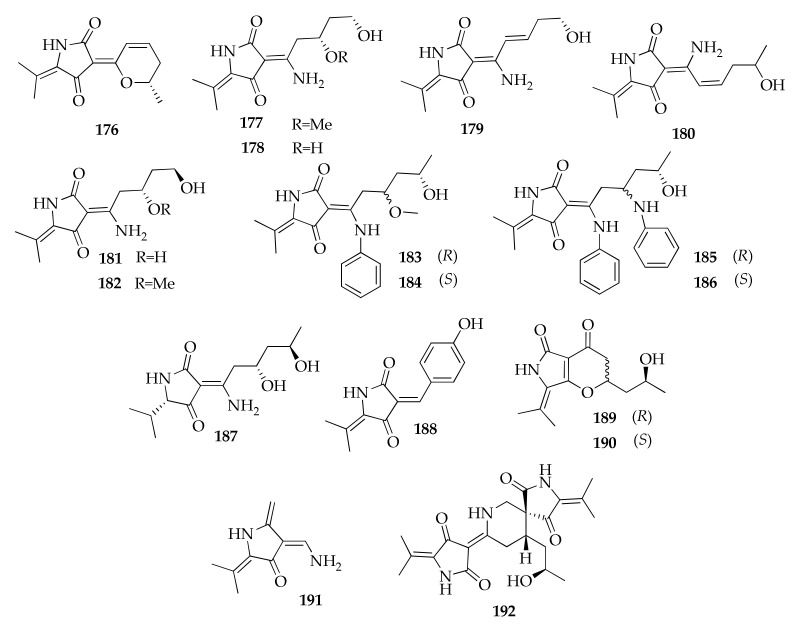
Structures of tetramic acids.

**Figure 28:**
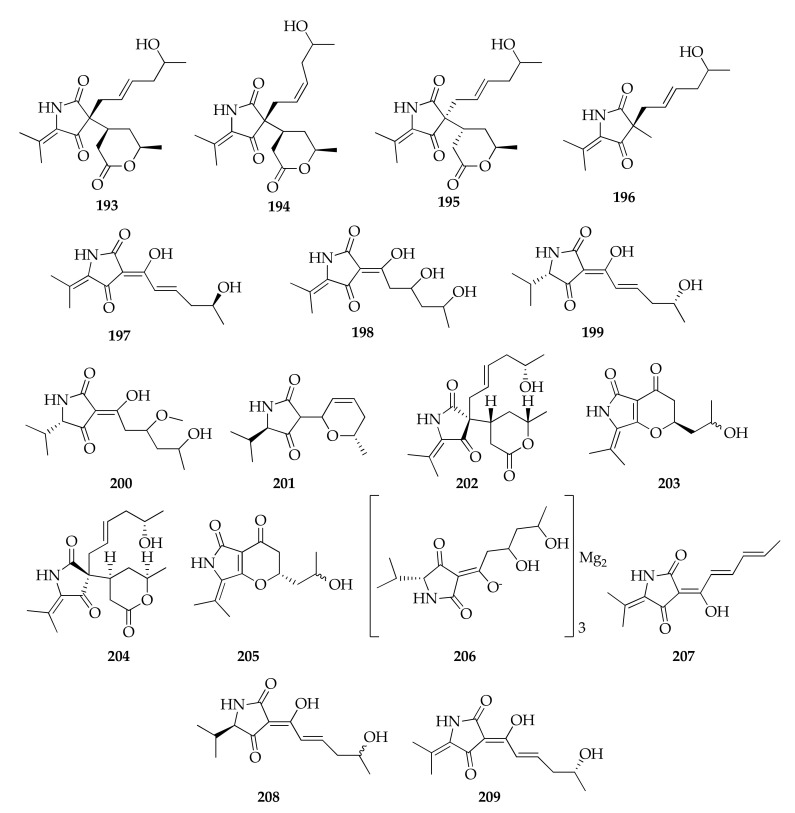
Structures of cladosporiumins.

Tetramic acids are compounds containing 2,4-pyrrolidinedione backbone obtained by the fusion of an amino acid with polyketide units. The series of cladosporimins and cladosins belong to this class, with the latter reported exclusively from *C. sphaerospermum* (Figure 27 and Figure 28). In fact, six novel cladosins, the structures of which are constituted by a tetramic acid core and 6(3)-enamino-8,10-dihydroxy or 6(3)-enamino-7(8)-en-10-ol side chains, named cladosins A–D (**177**–**180**) and F–G (**181**,**182**), were reported from a strain of *C. sphaerospermum* from sediments collected in the Pacific Ocean [55,56]. Each compound exists as two tautomeric forms differing in configuration of the enamine. Moreover, investigation into the fermentation extracts of another isolate of *C. sphaerospermum* from marine sediments led to the discovery of cladosins H–K (**183**–**186**) [89]. Finally, cladosins L–O (187, 189–191), together with another tetramic acid named cladodionen (176), were isolated from a strain of this species obtained from healthy bulbs of *Fritillaria unibracteata* var. *wabuensis* [63].

Two structurally different compounds were reported as cladosin L (**187**,**188**) in two papers published almost at the same time [60,63]. In fact, a second product labeled with this name (**188**) was identified from a *Hydractinia*-associated strain of *C. sphaerospermum*.

Even in the cladosporiumin series (Figure 28) there are some compounds that were given the same name because of the contemporaneous publication of work dealing with the structural identification of novel tetramic acids. In fact, Liang et al. [58] and Risher et al. [61] identified two tetramic acids continuing the series of cladosporiumins (**192**–**209**), and both research teams named their new compounds cladosporiumins I and J (**201**,**203**). Furthermore, cladosporiumin L (**206**), reported by Liang et al. [58], is a metal complex of tetramic acid. In fact, considering that the formation of metal complexes of tetramic acid derivatives (e.g., harzianic acid [128,129]) affect the chemical shifts of H-5 an N-methyl proton or NH, the authors can speculate that the structure of cladosporiumin L is a Mg_2_ complex. The authors also reported the structure of cladosporiumins F (**198**) and H (**200**) as their Na complexes.

### 2.26. Tropolones

**Figure 29:**
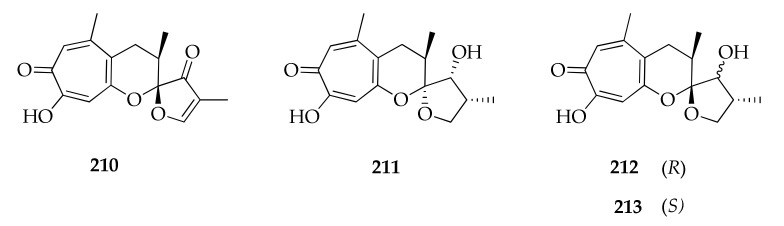
Structures of tropolones.

Malettinins A–C (**210–212**) were isolated and structurally elucidated from a marine strain of *Cladosporium* sp., along with the new malettinin E (**213**) (Figure 29) [95]. This represents the first isolation of tropolones from a fungus belonging to the genus *Cladosporium*. In fact, malettinins A–C were originally isolated from an unidentified fungus, which additionally produced a fourth metabolite, named malettinin D. This latter compound was not identified in the culture extracts of *Cladosporium* sp.; instead, its new 13-epimer was detected (**213**).

### 2.27. Volatile Terpenes

**Figure 30:**
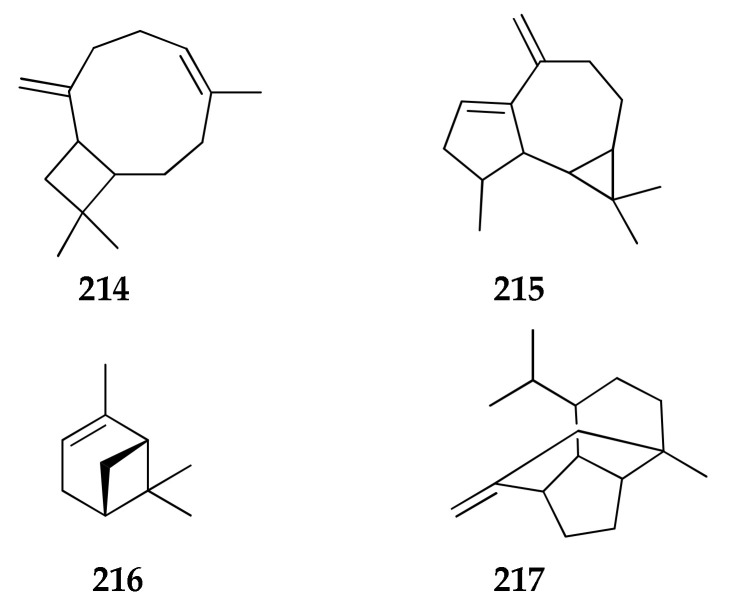
Structures of volatile terpenes.

An isolate of *C. cladosporioides* obtained from the rhizosphere of red pepper has been investigated for the production of volatile terpenes using solid phase microextraction (SPME) coupled to GC-MS (Figure 30) [38]. Identification of volatiles revealed mainly (−)-trans-caryophyllene, dehydroaromadendrene, α-pinene and (+)-sativene (**214**–**217**). In previous research on Plant Growth Promoting Rhizobacteria (PGPR) and Plant Growth Promoting Fungi (PGPF), it was reported that volatile terpenes play important chemo-ecological roles in the interactions between plants and their environments [130]. In fact, this strain seems to be able to improve the growth of tobacco seedlings and their root development through the production of volatile terpenes [38].

### 2.28. Xanthones

**Figure 31:**
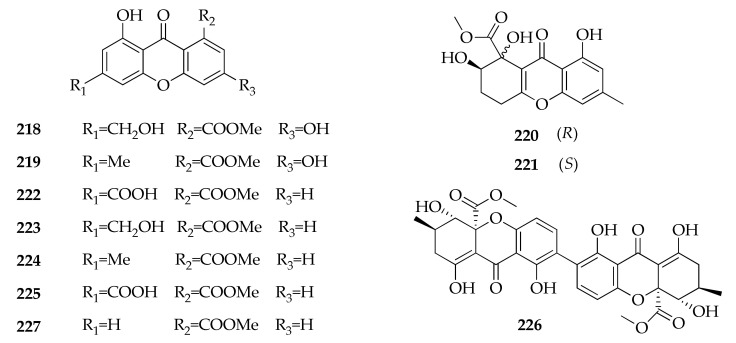
Structures of xanthones.

The class of xanthones includes compounds with a backbone designated as dibenzo-7-pyrone. A huge number of xanthones have been isolated from natural sources of higher plants, fungi, ferns, and lichens [131]. A strain of *C. halotolerans* symbiotic with the coral *Porites lutea* produces nine metabolites (**218**–**225**,**227**) belonging to this class (Figure 31) [43]. Furthermore, a dimeric tetrahydroxanthone (**226**), where two tetrahydroxanthone monomers are connected through a 2,2’-biphenol linkage, was also isolated from an endophytic strain of *Cladosporium* sp. [85].

### 2.29. Miscellaneous

**Figure 32:**
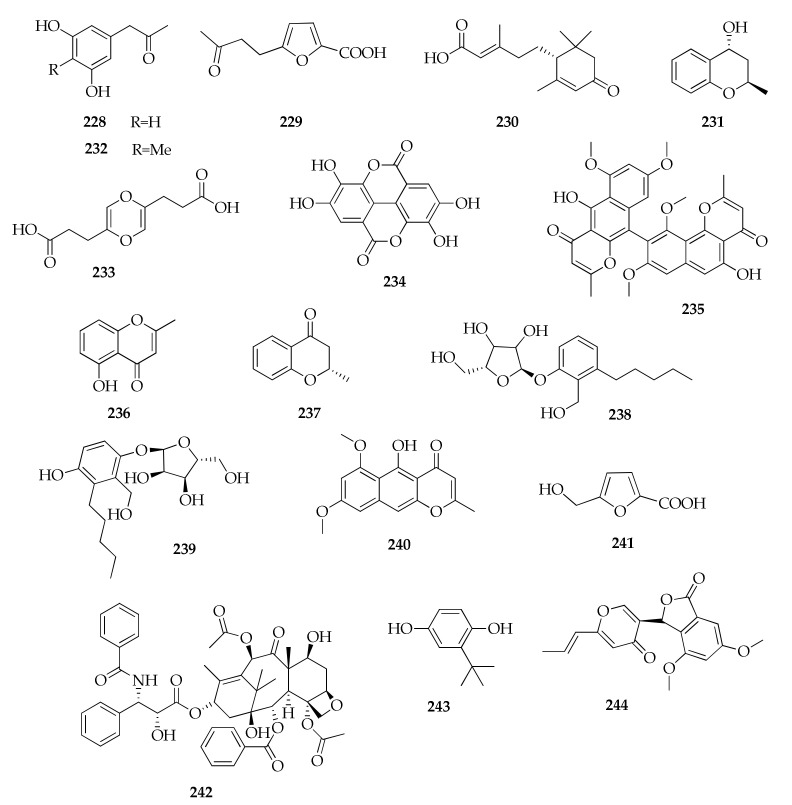
Structures of compounds from the group “miscellaneous”.

Finally, a number of products of *Cladosporium* are placed in a miscellaneous class because they have no structural affinity with previous groups (Figure 32). This is the case for a new abscisic acid analogue named cladosacid (**230**) [96], sumiki’s acids (**229**,**241**) [44], the new pentenoic acid derivative named 1,1′-dioxine-2,2′-dipropionic acid (**233**) [85] and two new ribofuranose phenol derivatives named 4-*O*-α-d-ribofuranose-3-hydroxymethyl-2-pentylphenol (**239**) and 4-*O*-α-d-ribofuranose-2-pentyl-3-phemethylol (**238**) [81,82].

## 3. Biological Activities of Secondary Metabolites

Most secondary metabolites reported in Table 3 have been investigated for biological properties, including antifungal, antibacterial cytotoxic and phytotoxic activities, which are summarized in Table 3. For some well-known compounds (e.g., the tetracyclic diterpenoid taxol and the funicone compound vermistatin), which have been extensively investigated and have been the subject of dedicated reviews [132], this table only considers data resulting from reports concerning the isolation of these compounds from *Cladosporium* strains.

Particularly valuable in the study of the bioactivities of natural products is the structure–activity relationship (SAR), but this aspect has only been taken into account in few research papers on *Cladosporium* compounds. An interesting evaluation of the relationships between structures and bioactivity was reported for cladosporin analogues by Wang et al. [25], who considered the presence of several essential positions in the chemical structures of these compounds that might be responsible for their antifungal activity. As a consequence, the antifungal activity of the parent compound seems to be influenced by the R configuration of C-6′. This configuration greatly decreased the antifungal activity of isocladosporin against the *Colletotrichum* species but slightly increased the antifungal activity against the *Phomopsis* species. Comparing the structures of cladosporin and 5′-hydroxyasperentin, the hydroxylation of the C-5′ position causes the loss of the antifungal activity against the *Colletotrichum* species and decreases the selectivity against the *Phomopsis* species. Comparison of 5′-hydroxyasperentin and the synthesized 6,5′-diacetyl derivative revealed that the replacement of the hydrogen in the hydroxyl group at C-6 and the hydrogen at C-5′ in the acetyl groups greatly increased selectivity toward the two *Phomopsis* species. Furthermore, the C-8 position also seems to be responsible for antifungal activity, demonstrated by the inactivity of asperentin-8-methyl ether against all the tested fungi [25].

Another interesting correlation between chemical structure and bioactivity arises from the investigation on the inhibitory activity of cladosporol towards β-l,3-glucan synthetase. In fact, the epoxy-alcohol moiety in cladosporol is very similar to another β-l,3-glucan biosynthesis inhibitor, (+)-isoepoxydon; hence, the epoxy-alcohol structure seems to play an important role in the inhibitory activity of β-l,3-glucan synthetase [26].

## 4. Conclusions

As resulting from the available information examined in this review, data concerning secondary metabolite production and properties in *Cladosporium* are notable in quantitative terms. Indeed, the biosynthetic aptitudes of these fungi are quite original, with several series of products for which they represent the only source known so far. At the same time, at least some strains have resulted in the sharing of genetic bases for producing bioactive compounds previously reported from other fungal genera, such as cytochalasin D, brefeldin A, vermistatin, zeaenol, the coniochaetones, the malettinins and the viridotoxins, or even from plants, such as the gibberellins, plumbagin and taxol, which represent a direction for their possible biotechnological exploitation.

Besides implications deriving from the bioactive properties of some valuable products, metabolomics has been also used as a tool for species description and discrimination in several fungal genera, such as *Penicillium, Talaromyces*, *Aspergillus* [133], *Alternaria* [134] and *Trichoderma* [135]. The great diversity of secondary metabolites reported from *Cladosporium* spp. could represent a notable base material for verifying if a similar approach can be consistent for this genus as well. So far, the number of isolates that have been examined in this respect is too small, with as many as 23 of them not having been ascribed to any definite species, and the only consistent aspect resulting from the analysis of the available literature is represented by the production of tetramic acids by *C. sphaerospermum*. However, it is to be expected that the likely accumulation of new reports based on accurate molecular identification referring to the most recent taxonomic schemes may pave the way to a chemotaxonomic perspective for *Cladosporium* too.

## Figures and Tables

**Table 1:** *Cladosporium* species/strains reported for production of secondary metabolites.

Species/Strain	Substrate	Location	Refs.
*C. cladosporioides*	-	-	[12]
*C. cladosporioides*	sediment of hypersaline lake	El Hamra, Egypt	[22]
*C. cladosporioides*	sponge (*Cliona* sp.)	Los Molles, Chile	[23]
*C. cladosporioides*	aphid (*Aphis craccivora*)	Egypt	[17,18]
*C. cladosporioides*	endophytic in *Zygophyllum mandavillei*	Al-Ahsa, Saudi Arabia	[24]
*C. cladosporioides*	endophytic in unspecified plant	Tifton, United States	[25]
*C. cladosporioides*	-	Japan	[26]
*C. cladosporioides*/CBUK20700	dead insect	Thailand	[27,28]
*C. cladosporioides*/FERMBP-1285	block fence	Osaka, Japan	[29,30]
*C. cladosporioides*/IPV-F167	-	Italy	[31]
*C. cladosporioides*/LWL5	endophytic in *Helianthus annuus*	Daegu, South Korea	[16]
*C. cladosporioides*/MA-299	endophytic in *Bruguiera gymnorrhiza*	Hainan, China	[32,33,34]
*C. cladosporioides*/MCCC3A00182	deep sea sediment	Pacific Ocean	[35]
*C. cladosporioides*/MUPGBIOCHEM-CC-07-2015	brown alga (*Sargassum wightii*)	Tamil Nadu, India	[21]
*C. cladosporioides*/NRRL5507	-	-	[36,37]
*C. cladosporioides*/CL-1	rhizosphere of red pepper	South Korea	[38]
*C. cladosporioides*/EN-399	red alga (*Laurencia okamurai*)	Qingdao, China	[39]
*C. cladosporioides*/HDN14-342	deep sea sediment	Indian Ocean	[40]
*C. colocasiae*/A801	endophytic in *Callistemon* *viminalis*	Guangzhou, China	[41]
*C. delicatulum*/EF33	endophytic in *Terminalia* *pallida*	Andhra Pradesh, India	[42]
*C. halotolerans*/GXIMD 02502	coral (*Porites lutea*)	Weizhou islands, China	[43]
*C. herbarum*	sponge (*Callyspongia aerizusa*)	Bali, Indonesia	[44,45]
*C. herbarum*/FC27P	endophytic in *Beta vulgaris*	Dublin, United States	[46]
*C. herbarum*/IFB-E002	endophytic in *Cynodon dactylon*	Yancheng reserve, China	[47]
*C. oxysporum*	endophytic in *Aglaia odorata*	Java, Indonesia	[14]
*C. oxysporum*	endophytic in *Alyxia reinwardtii*	Java, Indonesia	[48]
*C. oxysporum*/DH14	locust (*Oxya chinensis*)	Jinhua, China	[49]
*C. oxysporum/*HDN13-314	endophytic in *Avicennia marina*	Hainan, China	[50]
*C. oxysporum*/RM1	endophytic in *Moringa oleifera*	Tamil Nadu, India	[51]
*C. perangustum*/FS62	deep sea sediment	South China Sea	[52]
*C. phlei*/C-273 w	pathogenic on *Phleum pratense*	Hokkaido, Japan	[53]
*C. phlei*/CBS 358.69	pathogenic on *Phleum pratense*	Germany	[54]
*C. sphaerospermum*/2005-01-E3	deep sea sludge	Pacific Ocean	[55,56]
*C. sphaerospermum*/DK-1-1	endophytic in *Glycine max*	South Korea	[57]
*C. sphaerospermum*/EIODSF 008	deep sea sediment	Indian Ocean	[58]
*C. sphaerospermum*/L3P3	deep sea sediment	Mariana Trench	[59]
*C. sphaerospermum*/SW67	hydroid (*Hydractinia echinata*)	South Korea	[60,61,62]
*C. sphaerospermum*/WBS017	endophytic in *Fritillaria unibracteata* var. *wabuensis*	China	[63]
*C. tenuissimum*	soil	Karo-cho, Japan	[64]
*C. tenuissimum*/DMG 3	endophytic in *Swietenia mahagoni*	Sumatra, Indonesia	[65]
*C. tenuissimum*/ITT21	pine rust (*Cronartium flaccidum*)	Tuscany, Italy	[66]
*C. tenuissimum*/LR463	endophytic in *Maytenus hookeri*	Yunnan, China	[67]
*C. tenuissimum*/P1S11	endophytic in *Pinus wallichiana*	Kashmir, India	[68]
*C. uredinicola*	endophytic in *Psidium guajava*	São Carlos, Brazil	[69]
*C. velox*/TN-9S	endophytic in *Tinospora cordifolia*	Amritsar, India	[70]
*Cladosporium* sp.	sponge (*Niphates rowi*)	Gulf of Aqaba, Israel	[71]
*Cladosporium* sp./486	intertidal sediment	San Antonio Oeste, Argentina	[19]
*Cladosporium* sp./501-7w	-	Japan	[72,73,74]
*Cladosporium* sp./F14	sea water	Sai Kung, China	[13,75]
*Cladosporium* sp./HDN17-58	deep sea sediment	Pacific Ocean	[76]
*Cladosporium* sp./I(R)9-2	endophytic in *Quercus variabilis*	Nanjing, China	[77]
*Cladosporium* sp./IFB3lp-2	endophytic in *Rhizophora stylosa*	Hainan, China	[78]
*Cladosporium* sp./IFM 49189	-	Japan	[79,80]
*Cladosporium* sp./JJM22	endophytic in *Ceriops tagal*	Hainan, China	[81,82,83]
*Cladosporium* sp./JNU17DTH12-9-0	unknown	China	[84]
*Cladosporium* sp./JS1-2	endophytic in *Ceriops tagal*	Hainan, China	[85]
*Cladosporium* sp./KcFL6’	endophytic in *Kandelia candel*	Daya Bay, China	[86]
*Cladosporium* sp./KFD33	blood cockle	Hainan, China	[87]
*Cladosporium* sp./L037	brown alga (*Actinotrichia fragilis*)	Okinawa, Japan	[88]
*Cladosporium* sp./N5	red alga (*Porphyra yezoensis*)	Lianyungang, China	[15]
*Cladosporium* sp./OUCMDZ-1635	sponge	Xisha Islands, China	[89]
*Cladosporium* sp./RSBE-3	endophytic in *Rauwolfia serpentina*	Bangladesh	[90]
*Cladosporium* sp./SCNU-F0001	endophytic in unspecified mangrove	Zhuhai, China	[91]
*Cladosporium* sp./SCSIO z01	deep sea sediment	East China Sea	[92]
*Cladosporium* sp./TPU1507	unidentified sponge	Manado, Indonesia	[93]
*Cladosporium* sp./TZP-29	unidentified soft coral	Guangzhou, China	[94]
*Cladosporium* sp./KF501	water sample	Wadden Sea, Germany	[95]
*Cladosporium* sp./SCSIO z0025	deep sea sediment	Okinawa, Japan	[96]

**Table 2:** List of secondary metabolites produced by *Cladosporium* species. The Latin suffix “*bis*” is added when the same name has been previously introduced for another compound. The names of novel compounds are underlined.

Code	Name	Formula	Nominal Mass (U)	Refs.
**Alkaloids**				
**1**	Aspernigrin A	C_13_H_12_N_2_O_2_	228	[47]
**2**	Aspidospermidin-20-ol, 1-acetyl-17-methoxy	C_22_H_30_N_2_O_3_	370	[24]
**3**	Cladosin E	C_13_H_17_NO_4_	251	[55]
**4**	Cladosporine A	C_28_H_39_NO_2_	421	[84]
**5**	Cytochalasin D	C_30_H_37_NO_6_	507	[85]
**6**	2-Methylacetate-3,5,6-trimethylpyrazine	C_10_H_14_N_2_O_2_	194	[85]
**7**	Nonanal oxime	C_9_H_19_NO	157	[17]
**8**	2-Piperidinone methyl	C_6_H_11_NO	113	[17]
**Azaphilones**				
**9**	Bicyclic diol	C_11_H_14_O_4_	210	[52]
**10**	Lunatoic acid A	C_21_H_24_O_7_	388	[49]
**11**	Perangustol A	C_11_H_14_O_4_	210	[52]
**12**	Perangustol B	C_11_H_14_O_4_	210	[52]
**Benzofluoranthenones**				
**13**	(6b*S*,7*R*,8*S*)-4,9-Dihydroxy-7,8- dimethoxy-1,6b,7,8-tetra-hydro-2*H*- benzo[J]fluoranthen-3-one	C_23_H_22_O_4_	362	[27]
**14**	(6b*S*,7*R*)-4,9-Dihydroxy-7- methoxy-1,2,6b,7-tetrahydrobenzo[J]fluoranthen-3,8-dione	C_22_H_18_O_4_	346	[27]
**15**	(6b*R*,7*R*,8*S*)-7- Methoxy-4,8,9-trihydroxy- 1,6b,7,8-tetrahydro-2*H*- benzo[J]fluoranthen-3-one	C_22_H_20_O_4_	348	[27]
**16**	(6b*S*,7*R*,8*S*)-7- Methoxy-4,8,9-trihydroxy- 1,6b,7,8-tetrahydro-2*H*- benzo[J]fluoranthen-3-one	C_22_H_20_O_4_	348	[27,28]
**Benzopyranones**				
**17**	Coniochaetone A	C_13_H_10_O_4_	230	[43]
**18**	Coniochaetone B	C_13_H_12_O_4_	232	[43]
**19**	Coniochaetone K	C_13_H_10_O_6_	262	[43]
**Binaphthopyrones**				
**20**	Cladosporinone	C_33_H_30_O_14_	650	[22]
**21**	Viriditoxin	C_34_H_30_O_14_	662	[22]
**22**	Viriditoxin SC-28763	C_34_H_30_O_13_	646	[22]
**23**	Viriditoxin SC-30532	C_34_H_30_O_12_	630	[22]
**Butenolides and butanolides**				
**24**	Cladospolide F	C_12_H_22_O_4_	230	[94]
**25**	Cladospolide G	C_14_H_24_O_5_	272	[32]
**26**	Cladospolide H	C_12_H_18_O_3_	210	[32]
**27**	ent-Cladospolide F	C_12_H_22_O_4_	230	[32]
**28**	11-Hydroxy-γ-dodecalactone	C_12_H_20_O_3_	212	[94]
**29**	iso-Cladospolide B	C_12_H_20_O_4_	228	[32,44,50,67,71,94]
**Cinnamic acid derivatives**				
**30**	Chlorogenic acid	C_16_H_18_O_9_	354	[70]
**31**	Caffeic acid	C_9_H_8_O_4_	180	[70]
**32**	Coumaric acid	C_9_H_8_O_3_	164	[70]
**Citrinin derivatives**				
**33**	Citrinin H1	C_25_H_30_O_7_	442	[85]
**34**	Cladosporin A	C_13_H_15_NO_3_	233	[92]
**35**	Cladosporin B	C_13_H_15_NO_3_	233	[92]
**36**	Cladosporin C	C_14_H_16_O_4_	248	[92]
**37**	Cladosporin D	C_12_H_16_O_4_	224	[92]
**Coumarins and Isocoumarins**				
**38**	Asperentin-8-methyl ether (= cladosporin-8-methyl ether)	C_17_H_22_O_6_	322	[25]
**39**	Cladosporin (= asperentin)	C_16_H_20_O_5_	292	[12,24,25,36,37]
**40**	5′-Hydroxyasperentin	C_16_H_20_O_6_	308	[24,25]
**41**	7-Hydroxy-4-methoxy-5-methylcoumarin	C_11_H_10_O_4_	206	[47]
**42**	Isocladosporin	C_16_H_20_O_5_	292	[24,25,37]
**43**	Kotanin	C_24_H_22_O_8_	438	[47]
**44**	Orlandin	C_22_H_18_O_8_	410	[47]
**45**	Phomasatin	C_10_H_8_O_5_	208	[35]
**46**	Umbelliferone	C_9_H_6_O_3_	162	[70]
**Cyclohexene derivatives**				
**47**	Cladoscyclitol A	C_12_H_20_O_5_	244	[82]
**48**	Cladoscyclitol B	C_13_H_22_O_7_	290	[82]
**49**	Cladoscyclitol C	C_12_H_22_O_4_	230	[82]
**50**	Cladoscyclitol D	C_12_H_22_O_5_	246	[82]
**Despsides**				
**51**	3-Hydroxy-2,4,5-trimethylphenyl 2,4-dihydroxy-3,6-dimethylbenzoate	C_18_H_20_O_5_	316	[69,98]
**52**	3-Hydroxy-2,4,5-trimethylphenyl 4-[(2,4-dihydroxy-3,6-dimethylbenzoyl)oxy]-2-hydroxy-3,6-dimethylbenzoate	C_27_H_28_O_8_	480	[69,98]
**53**	3-Hydroxy-2,5-dimethylphenyl 2,4-dihydroxy- 3,6-dimethylbenzoate	C_17_H_18_O_5_	302	[69,98]
**54**	3-Hydroxy-2,5-dimethylphenyl 4-[(2,4-dihydroxy-3,6-dimethylbenzoyl)oxy]-2-hydroxy-3,6-dimethylbenzoate	C_26_H_26_O_8_	466	[69,98]
**Diketopiperazines**				
**55**	(3*R*,8a*R*)-*Cyclo*(leucylprolyl)	C_11_H_18_N_2_O_2_	210	[17]
**56**	(3*S*,8a*S*)-*Cyclo*(leucylprolyl)	C_11_H_18_N_2_O_2_	210	[17]
**57**	(3*R*,8a*R*)-*Cyclo*(phenylalanylprolyl)	C_14_H_16_N_2_O_2_	244	[17]
**58**	(3*S*,8a*S*)-*Cyclo*(phenylalanylprolyl)	C_14_H_16_N_2_O_2_	244	[17]
**Flavonoids**				
**59**	(2*S*)-7,4′-Dihydroxy-5-methoxy-8-(γ,γ-dimethylallyl)-flavanone	C_21_H_22_O_5_	354	[93]
**60**	Catechin	C_15_H_14_O_6_	290	[70]
**61**	Epicatechin	C_15_H_14_O_6_	290	[70]
**Gibberelines**				
**62**	GA3	C_19_H_22_O_6_	346	[57]
**63**	GA4	C_19_H_24_O_5_	332	[57]
**64**	GA5	C_19_H_22_O_5_	330	[57]
**65**	GA7	C_19_H_22_O_5_	330	[57]
**66**	GA15	C_20_H_26_O_4_	330	[57]
**67**	GA19	C_20_H_26_O_6_	362	[57]
**68**	GA24	C_20_H_26_O_5_	346	[57]
**Fusicoccane diterpene glycosides**				
**69**	Cotylenin A	C_33_H_50_O_11_	622	[72,73,74]
**70**	Cotylenin B	C_33_H_51_ClO_11_	659	[73,74]
**71**	Cotylenin C	C_33_H_52_O_11_	624	[73]
**72**	Cotylenin D	C_33_H_52_O_12_	640	[73]
**73**	Cotylenin E	C_28_H_46_O_9_	526	[73]
**Lactones**				
**74**	Cladosporactone A	C_10_H_12_O_4_	196	[35]
**75**	Cladosporamide A	C_14_H_11_NO_5_	273	[93]
**76**	5-Decanolide	C_10_H_18_O_2_	170	[17]
**77**	Herbaric acid	C_10_H_8_O_6_	224	[45]
**78**	Isochracinic acid	C_10_H_8_O_5_	208	[35]
**Macrolides**				
**79**	Brefeldin A	C_16_H_24_O_4_	280	[77]
**80**	Cladocladosin A	C_13_H_18_O_3_	222	[34]
**81**	Cladospamide A	C_13_H_20_N_2_O_4_	268	[91]
**82**	Cladospolide A	C_12_H_20_O_4_	228	[41,64,67,78,91]
**83**	Cladospolide B	C_12_H_20_O_4_	228	[44,64,67,78]
**84**	Cladospolide C	C_12_H_20_O_4_	228	[64]
**85**	4,5-Dihydroxy-12-methyloxacyclododecan-2-one	C_12_H_22_O_4_	230	[67]
**86**	(6*R*,12*S*)-6-Hydroxy-12-methyl- oxacyclodoecane-2,5-dione	C_12_H_20_O_4_	228	[67]
**87**	(10*S*,12*S*)-10-Hydroxy-12-methyloxacyclododecane-2,5-dione	C_12_H_20_O_4_	228	[67]
**88**	4-Hydroxy-12-methyloxacyclododecane-2,5,6-trione	C_12_H_18_O_5_	242	[41]
**89**	(*E*)-(3*R*,6*S*)-6-Hydroxy-12-methyl-2,5-dioxooxacyclododecan-3-yl 4,11-dihydroxydodec-2-enoate	C_24_H_40_O_8_	456	[78]
**90**	5*R*-Hydroxyrecifeiolide	C_12_H_20_O_3_	212	[32]
**91**	5*S*-Hydroxyrecifeiolide	C_12_H_20_O_3_	212	[32]
**92**	12-Methyloxacyclododecane-2,5,6-trione	C_12_H_18_O_4_	226	[41]
**93**	Methyl 2-(((4*R*,6*S*,12*R*)-6-hydroxy-12-methyl-2,5-dioxooxacyclododecan-4-yl)thio)acetate	C_15_H_24_O_6_S	332	[78]
**94**	5Z-7-Oxozeaenol	C_19_H_22_O_7_	362	[49]
**95**	Pandangolide 1	C_12_H_20_O_5_	244	[32,41,48,71,78]
**96**	Pandangolide 1a	C_12_H_20_O_5_	244	[71,78]
**97**	Pandangolide 2	C_14_H_22_O_6_S	318	[44,78]
**98**	Pandangolide 3	C_16_H_26_O_7_S	362	[33,44,50,78]
**99**	Pandangolide 4	C_24_H_38_O_8_S	486	[44]
**100**	Patulolide B	C_13_H_20_O_3_	224	[41]
**101**	Sporiolide A	C_19_H_24_O_6_	348	[88]
**102**	Sporiolide B	C_14_H_24_O_4_	256	[88]
**103**	Thiocladospolide A	C_16_H_26_O_6_S	346	[33,50]
**104**	Thiocladospolide B	C_16_H_24_O_7_S	360	[33]
**105**	Thiocladospolide C	C_15_H_22_O_6_S	330	[33]
**106**	Thiocladospolide D	C_16_H_28_O_7_S	364	[33]
**107**	Thiocladospolide E	C_14_H_26_O_5_S	306	[91]
**108**	Thiocladospolide F	C_16_H_28_O_5_S	332	[34]
**109**	Thiocladospolide F *bis*	C_24_H_38_O_8_S	486	[50]
**110**	Thiocladospolide G	C_16_H_28_O_6_S	348	[34]
**111**	Thiocladospolide G *bis*	C_15_H_24_O_7_S	348	[50]
**112**	Thiocladospolide H	C_15_H_24_O_6_S	332	[50]
**113**	Thiocladospolide I	C_27_H_44_O_10_S	560	[50]
**114**	Thiocladospolide J	C_27_H_42_O_10_S	558	[50]
**115**	Zeaenol	C_19_H_24_O_7_	364	[49]
**Naphthalene derivatives**				
**116**	Cladonaphchrom A	C_22_H_22_O_4_	350	[83]
**117**	Cladonaphchrom B	C_22_H_22_O_4_	350	[83]
**118**	1,8-Dimethoxynaphthalene	C_12_H_12_O_2_	188	[81,83]
**119**	8-Methoxynaphthalen-1-ol	C_11_H_10_O_2_	174	[83]
**Naphtalenones**				
**120**	Altertoxin XII	C_20_H_18_O_4_	322	[87]
**121**	Cladosporol A	C_20_H_16_O_6_	352	[26,66,86]
**122**	Cladosporol B	C_20_H_14_O_6_	350	[66]
**123**	Cladosporol C	C_20_H_18_O_5_	338	[35,39,40,66,85,86]
**124**	Cladosporol D	C_20_H_18_O_6_	354	[66,86]
**125**	Cladosporol E	C_20_H_18_O_7_	370	[40,66,85]
**126**	Cladosporol F	C_21_H_20_O_5_	352	[39,40]
**127**	Cladosporol G	C_20_H_17_ClO_6_	388	[40]
**128**	Cladosporol G *bis*	C_21_H_20_O_5_	352	[39]
**129**	Cladosporol H	C_20_H_16_O_5_	336	[39]
**130**	Cladosporol I	C_20_H_18_O_5_	338	[39,92]
**131**	Cladosporol J	C_20_H_18_O_5_	338	[39]
**132**	Cladosporone A	C_20_H_16_O_6_	352	[86]
**133**	Clindanone A	C_22_H_18_O_7_	394	[40]
**134**	Clindanone B	C_22_H_18_O_7_	394	[40]
**135**	(3*R*,4*R*)-3,4-Dihydro-3,4,8-trihydroxy-1(2*H*)-napthalenone	C_10_H_10_O_4_	194	[81]
**136**	4,8-Dihydroxy-1-tetralone	C_10_H_10_O_3_	178	[52]
**137**	(3*S*)-3,8-Dihydroxy-6,7-dimethyl-α-tetralone	C_12_H_14_O_3_	206	[81]
**138**	Isosclerone	C_10_H_10_O_3_	178	[40]
**139**	Scytalone	C_10_H_10_O_4_	194	[68]
**140**	(−)-(4*R*)-Regiolone	C_10_H_10_O_3_	178	[81]
**141**	(−)-*trans*-(3*R*,4*R*)-3,4,8-Trihydroxy-6,7-dimethyl-3,4- dihydronaphtha- len-1(2*H*)-one	C_12_H_14_O_4_	222	[81]
**Naphthoquinones and anthraquinones**				
**142**	Anhydrofusarubin	C_15_H_12_O_6_	288	[90]
**143**	Methyl ether of fusarubin	C_16_H_16_O_7_	320	[90]
**144**	Plumbagin	C_11_H_8_O_3_	188	[42]
**Perylenequinones**				
**145**	Altertoxin VIII	C_20_H_16_O_3_	304	[87]
**146**	Altertoxin IX	C_20_H_18_O_2_	290	[87]
**147**	Aterotoxin X	C_20_H_18_O_2_	290	[87]
**148**	Altertoxin XI	C_21_H_20_O_2_	304	[87]
**149**	Calphostin A (= UCN-1028A)	C_44_H_38_O_12_	758	[29,30]
**150**	Calphostin B	C_37_H_34_O_11_	654	[30]
**151**	Calphostin C (= Cladochrome E)	C_44_H_38_O_14_	790	[30,31]
**152**	Calphostin D (= ent-isophleinchrome)	C_30_H_30_O_10_	550	[30,46]
**153**	Calphostin I (= Cladochrome D)	C_44_H_38_O_15_	806	[30,31]
**154**	Phleichrome	C_30_H_30_O_10_	550	[53,54,99]
**Pyrones**				
**155**	Citreoviridin A	C_23_H_30_O_6_	402	[45]
**156**	Herbarin A	C_12_H_12_O_5_	236	[45]
**157**	Herbarin B	C_10_H_10_O_5_	210	[45]
**158**	5-Hydroxy-2-oxo-2*H*-piran-4-yl) methyl acetate	C_8_H_8_O_5_	184	[65]
**Seco acids**				
**159**	Cladospolide E	C_12_H_20_O_4_	228	[94]
**160**	11-Hydroxy-4,5-dioxododecanoic acid	C_12_H_20_O_5_	244	[78]
**161**	seco-Patulolide A	C_12_H_20_O_4_	228	[94]
**162**	seco-Patulolide C	C_12_H_22_O_4_	230	[33,41,50,94]
**163**	(3*S*,5*S*, 11*S*)-Trihydroxydodecanoic acid	C_12_H_24_O_5_	248	[68]
**Sterols**				
**164**	Cladosporide A	C_25_H_40_O_3_	388	[79]
**165**	Cladosporide B	C_25_H_38_O_3_	386	[80]
**166**	Cladosporide C	C_25_H_40_O_3_	388	[80]
**167**	Cladosporide D	C_25_H_38_O_3_	386	[80]
**168**	Cladosporisteroid B	C_21_H_30_O_3_	330	[35]
**169**	(22*E*,24*R*)-3β,5α-Dihydroxyergosta-7,22-dien-6-one	C_28_H_44_O_3_	428	[35]
**170**	Ergosterol	C_28_H_44_O	396	[79]
**171**	3α-Hydroxy-pregn-7-ene-6,20-dione	C_21_H_30_O_3_	330	[62]
**172**	23,24,25,26,27-Pentanorlanost-8-ene-3β,22-diol	C_28_H_42_O_5_	458	[79]
**173**	Peroxyergosterol (= (22*E*)-5α,8α-epidioxyergosta-6,22-dien-3β-ol)	C_28_H_44_O_3_	428	[23,35,79]
**174**	3β,5α,6β-Trihydroxyergosta-7,22-diene	C_29_H_48_O_3_	444	[47]
**175**	3β,5α,9α-Trihydroxy- (22*E*,24*R*)-ergosta-6-one	C_28_H_44_O_4_	444	[35]
**Tetramic acids**				
**176**	Cladodionen	C_13_H_15_NO_3_	233	[58,63,89,96]
**177**	Cladosin A	C_13_H_20_N_2_O_4_	268	[55]
**178**	Cladosin B	C_12_H_18_N_2_O_4_	254	[55,60,63]
**179**	Cladosin C	C_12_H_16_N_2_O_3_	236	[55,60,63]
**180**	Cladosin D	C_13_H_18_N_2_O_3_	250	[55]
**181**	Cladosin F	C_12_H_18_N_2_O_4_	254	[56,60,63]
**182**	Cladosin G	C_13_H_20_N_2_O_4_	268	[56]
**183**	Cladosin H	C_20_H_26_N_2_O_4_	358	[89]
**184**	Cladosin I	C_20_H_26_N_2_O_4_	358	[89]
**185**	Cladosin J	C_25_H_29_N_3_O_3_	419	[89]
**186**	Cladosin K	C_25_H_29_N_3_O_3_	419	[89]
**187**	Cladosin L	C_13_H_22_N_2_O_4_	270	[60]
**188**	Cladosin L *bis*	C_14_H_13_NO_3_	243	[63]
**189**	Cladosin M	C_13_H_17_NO_4_	251	[63]
**190**	Cladosin N	C_13_H_17_NO_4_	251	[63]
**191**	Cladosin O	C_9_H_12_N_2_O	164	[63]
**192**	Cladosporicin A	C_21_H_27_N_3_O_5_	401	[61]
**193**	Cladosporiumin A	C_19_H_27_NO_5_	349	[59]
**194**	Cladosporiumin B	C_19_H_27_NO_5_	349	[59]
**195**	Cladosporiumin C	C_19_H_27_NO_5_	349	[59]
**196**	Cladosporiumin D	C_14_H_21_NO_3_	251	[59]
**197**	Cladosporiumin E	C_13_H_17_NO_4_	251	[58,59]
**198**	Cladosporiumin F	C_13_H_19_NO_5_	269	[59]
**199**	Cladosporiumin G	C_13_H_19_NO_4_	253	[58,59]
**200**	Cladosporiumin H	C_14_H_23_NO_5_	285	[59]
**201**	Cladosporiumin I	C_13_H_19_NO_3_	237	[58]
**202**	Cladosporiumin I *bis*	C_19_H_27_NO_5_	**349**	[61]
**203**	Cladosporiumin J	C_13_H_17_NO_4_	251	[58]
**204**	Cladosporiumin J *bis*	C_19_H_27_NO_5_	349	[61]
**205**	Cladosporiumin K	C_13_H_17_NO_4_	**251**	[58]
**206**	Cladosporiumin L	(C_13_H_20_NO_5_)_3_Mg_2_	889	[58]
**207**	Cladosporiumin M	C_13_H_15_NO_3_	233	[58]
**208**	Cladosporiumin N	C_13_H_19_NO_4_	253	[58]
**209**	Cladosporiumin O	C_13_H_17_NO_4_	251	[58]
**Tropolones**				
**210**	Malettinin A	C_16_H_16_O_5_	288	[95]
**211**	Malettinin B	C_16_H_20_O_5_	292	[95]
**212**	Malettinin C	C_16_H_20_O_5_	292	[95]
**213**	Malettinin E	C_16_H_20_O_5_	292	[95]
**Volatile terpenes**				
**214**	(−)-*trans*-Caryophyllene	C_15_H_24_	204	[38]
**215**	Dehydro aromdendrene	C_15_H_22_	202	[38]
**216**	α-Pinene	C_10_H_16_	136	[38]
**217**	(+)-Sativene	C_15_H_24_	204	[38]
**Xanthones**				
**218**	Conioxanthone A	C_16_H_12_O_7_	316	[43]
**219**	3,8-Dihydroxy-6-methyl-9-oxo-9*H*-xanthene-1-carboxylate	C_16_H_12_O_6_	300	[43]
**220**	α-Diversonolic ester	C_16_H_16_O_7_	320	[43]
**221**	β-Diversonolic ester	C_16_H_16_O_7_	320	[43]
**222**	8-Hydroxy-6-methylxanthone-1-carboxylic acid	C_15_H_12_O_5_	272	[43]
**223**	Methyl 8-hydroxy-6-(hydroxymethyl)- 9-oxo-9*H*-xanthene-1-carboxylate	C_16_H_12_O_6_	300	[43]
**224**	Methyl 8-hydroxy-6-methyl-9-oxo-9*H*-xanthene-1- carboxylate	C_16_H_12_O_5_	284	[43]
**225**	8-(Methoxycarbonyl)-1-hydroxy-9-oxo-9*H*-xanthene-3-carboxylic acid	C_16_H_10_O_7_	314	[43]
**226**	Secalonic acid D	C_32_H_30_O_14_	638	[85]
**227**	Vertixanthone	C_15_H_10_O_5_	270	[43]
**Miscellaneous**				
**228**	α-Acetylorcinol	C_9_H_10_O_3_	166	[52]
**229**	Acetyl Sumiki’s acid	C_9_H_10_O_4_	182	[44]
**230**	Cladosacid	C_15_H_22_O_3_	250	[96]
**231**	(2R*,4R*)-3,4-dihydro-5-methoxy-2-methyl-1(2*H*)-benzopyran-4-ol	C_10_H_12_O_2_	164	[81]
**232**	1-(3,5-Dihydroxy-4-methylphenyl)propan-2-one	C_10_H_12_O_3_	180	[52]
**233**	1,1′-Dioxine-2,2′-dipropionic acid	C_10_H_12_O_6_	228	[85]
**234**	Ellagic acid	C_14_H_6_O_8_	302	[70]
**235**	Fonsecinone A	C_32_H_26_O_10_	570	[47]
**236**	5-Hydroxy-2-methyl-4*H*-chromen-4-one	C_10_H_8_O_3_	176	[83]
**237**	(2*S*)-5-Hydroxy-2-methyl-chroman-4-one	C_10_H_10_O_2_	162	[81,83]
**238**	4-*O*-α -d-Ribofuranose-2-pentyl-3-phemethylol	C_17_H_26_O_6_	326	[82]
**239**	4-*O*-α-d-Ribofuranose-3-hydroxymethyl-2-pentylphenol	C_17_H_26_O_7_	342	[81]
**240**	Rubrofusarin B	C_16_H_14_O_5_	286	[47]
**241**	Sumiki’s acid	C_6_H_6_O_4_	142	[44]
**242**	Taxol	C_47_H_51_NO_14_	853	[51]
**243**	tert-Butylhydroquinone	C_10_H_14_O_2_	166	[70]
**244**	Vermistatin	C_18_H_16_O_6_	328	[85]

**Table 3:** Bioactivities of secondary metabolites produced by the *Cladosporium* species.

Name (Code)	Biological Activity	Concentration	Results	Ref.
**Alkaloids**				
Aspidospermidin-20-ol, 1-acetyl-17-methoxy (**2**)	Antimicrobial	125 µg mL^−1^;	*Xanthomonas oryzae* (MIC);	[24]
		62.50 µg mL^−1^;	*Pseudomonas syringae* (MIC);	
		320.5 µg mL^−1^	*Aspergillus flavus* (MIC)	
Cladosporine A (**4**)	Antimicrobial	4 μg mL^−1^;	*Staphylococcus aureus* (MIC);	[84]
		16 μg mL^−1^	*Candida albicans* (MIC)	
Cytochalasin D (**5**)	Antibacterial	25 μg mL^–1^	*S. aureus* (MIC)	[85]
2-Methylacetate-3,5,6-trimethylpyrazine (**6**)	Antibacterial	12.5 μg mL^–1^	*S. aureus* (MIC)	[85]
**Azaphilones**				
Lunatoic acid A (**10**)	Phytotoxic	100 μg mL^–1^	*Brassica rapa*; *Sorghum durra*; *Brassica campestris*; *Capsicum annuum*; *Raphanus sativus*	[49]
**Benzofluoranthenones**				
(6b*S*,7*R*,8*S*)-4,9-Dihydroxy-7,8-dimethoxy-1,6b,7,8-tetra-hydro-2*H*-benzo[J]fluoranthen-3-one (**13**)	Inhibition of anti-CD28-induced IL2	2.4 µM	IC_50_	[27]
(6b*R*,7*R*,8*S*)-7-Methoxy-4,8,9-trihydroxy-1,6b,7,8-tetrahydro-2*H*-benzo[J]fluoranthen-3-one (**15**)	Inhibition of anti-CD28-induced IL2	2.5 µM	IC_50_	[27]
	Abl tyrosine kinase	0.76 µM	IC_50_	
(6b*S*,7*R*,8*S*)-7-Methoxy-4,8,9-trihydroxy-1,6b,7,8-tetrahydro-2*H*-benzo[J]fluoranthen-3-one (**16**)	Inhibition of anti-CD28-induced IL2	0.4 µM	IC_50_	[27]
	Abl tyrosine kinase	0.06 µM	IC_50_	
**Benzopyranones**				
Coniochaetone A (**17**)	Cytotoxic	10 µM	22RV1 (67.4%), C4-2B (13.87%), RWPE-1 (17.3%)	[43]
Coniochaetone B (**18**)	Cytotoxic	10 µM	22RV1 (32.7%), C4-2B (2.9%), RWPE-1 (19.7%)	[43]
Coniochaetone K (**19**)	Cytotoxic	10 µM	22RV1 (64.6%), C4-2B (7.2%), RWPE-1 (11.7%)	[43]
**Binaphthopyrones**				
Cladosporinone (**20**)	Cytotoxic	53.7 µM	L5178Y (IC_50_)	[22]
	Antibacterial	64 µg mL^−1^	*S. aureus* (MIC)	
Viriditoxin (**21**)	Cytotoxic	0.1 µM	L5178Y (IC_50_)	[22]
	Antibacterial	0.015 µg mL^−1^	*S. aureus* (MIC)	
Viriditoxin SC-28763 (**22**)	Cytotoxic	0.25 µM	L5178Y (IC_50_)	[22]
	Antibacterial	2 µg mL^−1^	*S. aureus* (MIC)	
Viriditoxin SC-30532 (**23**)	Antibacterial	16 µg mL^−1^	*S. aureus* (MIC)	[22]
**Butenolides and butanolides**				
Cladospolide F (**24**)	Lipid accumulation	10 µM	Oleic acid	[94]
Cladospolide G (**25**)	Antimicrobial	32 µg mL^−1^;	*E. coli* (MIC);	[32]
		1 µg mL^−1^;	*Glomerella cingulata* (MIC);	
		32 µg mL^−1^;	*Bipolaris sorokiniana* (MIC);	
		1 µg mL^−1^	*Fusarium oxysporum* f. sp. *cucumerinum* (MIC)	
ent-Cladospolide F (**27**)	Antibacterial	8 µg mL^−1^;	*S. aureus* (MIC);	[32]
		16 µg mL^−1^;	*Edwardsiella ictarda* (MIC);	
		64 µg mL^−1^	*P. aeruginosa* (MIC)	
	Acetylcholinesterase	40.46 µM	IC_50_	
11-Hydroxy-γ-dodecalactone (**28**)	Lipid accumulation	10 µM	Oleic acid	[94]
iso-Cladospolide B (**29**)	Antimicrobial	32 µg mL^−1^;	*E. coli* (MIC);	[32]
		32 µg mL^−1^;	*Edwardsiella tarda* (MIC);	
		16 µg mL^−1^;	*E. ictarda* (MIC);	
		64 µg mL^−1^	*G. cingulata* (MIC)	
	Antimicrobial	16 µg mL^−1^;	*E. tarda* (MIC);	[50]
		8 µg mL^−1^;	*E. ictarda* (MIC);	
		8 µg mL^−1^;	*Clematis mandshurica Miura* (MIC);	
		16 µg mL^−1^;	*Colletotrichum gloeosporioides* (MIC);	
		32 µg mL^−1^;	*B. sorokiniana* (MIC);	
		32 µg mL^−1^	*F. oxysporum* f. sp. *cucumerinum* (MIC)	
**Citrinin dervatives**				
Citrinin H1 (**33**)	Antibacterial	6.25 μg mL^−1^;	*S. aureus* (MIC);	[85]
		12.5 μg mL^−1^;	*E. coli* (MIC);	
		12.5 μg mL^−1^	*B. cereus* (MIC)	
Cladosporin A (**34**)	Toxic	72.0 µM	brine shrine nauplii (IC_50_)	[92]
Cladosporin B (**35**)	Toxic	81.7 µM	brine shrine nauplii (IC_50_)	[92]
Cladosporin C (**36**)	Toxic	49.9 µM	brine shrine nauplii (IC_50_)	[92]
Cladosporin D (**37**)	Antioxidant	16.4 µM	DPPH radicals (IC_50_)	[92]
	Toxic	81.4 µM	brine shrine nauplii (IC_50_)	
**Coumarins and isocoumarins**				
Cladosporin (**39**)	Antimicrobial	75 µg mL^−1^;	dermatophytes (100%);	[12]
		40 µg mL^−1^	spore germination of *Penicillium* sp. (100%) and *Aspergillus* sp. (100%)	
		30 µM	*Colletotrichum acutatum* (92.7%), *Colletotrichum fragariae* (90.1%), *C. gloeosporioides* (95.4%), *Plasmopara viticola* (79.9%)	[25]
		500 µg mL^−1^;	*X. oryzae* (MIC), *A. flavus* (MIC);	[24]
		62.50 µg mL^−1^	*Fusarium solani* (MIC)	
	Phytotoxic	10^−3^ M	etiolated wheat (81%)	[37]
5′-Hydroxyasperentin (**40**)	Antimicrobial	15.62 µg mL^−1^;	*X. oryzae* (MIC);	[24]
		62.50 µg mL^−1^;	*P. syringae* (MIC);	
		15.62 µg mL^−1^;	*A. flavus* (MIC);	
		7.81 µg mL^−1^	*F. solani* (MIC)	
		30 µM	*P. viticola* (53.9%), *Phomopsis obscurans* (25.6%)	[25]
Isocladosporin (**41**)	Antimicrobial	30 µM	*C. fragariae* (50.4%), *C. gloeosporioides* (60.2%), *P. viticola* (83.0%)	[25]
		15.62 µg mL^−1^;	*X. oryzae* (MIC), *P. syringae* (MIC);	[24]
		125 µg mL^−1^;	*A. flavus* (MIC);	
		62.50 µg mL^−1^	*F. solani* (MIC)	
	Phytotoxic	10^−3^ M	etiolated wheat (100%)	[37]
**Cyclohexene derivatives**				
Cladoscyclitol B (**48**)	Inhibition of α-glucosidase	2.95 µM	IC_50_	[82]
**Depsides**				
3-Hydroxy-2,4,5-trimethylphenyl 4-[(2,4-dihydroxy-3,6-dimethylbenzoyl)oxy]-2-hydroxy-3,6-dimethylbenzoate (**51**)	Antimicrobial	25 µg mL^−1^;	*B. subtilis* (bacteriostatic);	[69,98]
		25 µg mL^−1^;	*P. aeruginosa* (bacteriostatic);	
		250 µg mL^−1^;	*E. coli* (bacteriostatic);	
		250 µg mL^−1^	*S. aureus* (bacteriostatic)	
3-Hydroxy-2,5-dimethylphenyl 2,4-dihydroxy-3,6-dimethylbenzoate (**53**)	Antimicrobial	25 µg mL^−1^;	*B. subtilis* (MIC);	[69,98]
		25 µg mL^−1^;	*P. aeruginosa* (MIC);	
		250 µg mL^−1^;	*E. coli* (MIC);	
		250 µg mL^−1^;	*S. aureus* (MIC)	
3-Hydroxy-2,5-dimethylphenyl 4-[(2,4-dihydroxy-3,6-dimethylbenzoyl)oxy]-2-hydroxy-3,6-dimethylbenzoate (**54**)	Antimicrobial	250 µg mL^−1^;	*B. subtilis* (bacteriostatic);	[69,98]
		250 µg mL^−1^;	*P. aeruginosa* (bacteriostatic);	
		250 µg mL^−1^;	*E. coli* (bacteriostatic);	
		250 µg mL^−1^	*S. aureus* (bacteriostatic)	
**Flavonoids**				
(2S)-7,4′-Dihydroxy-5-methoxy-8-(γ,γ-dimethylallyl)- flavanone (**59**)	Enzymatic inhibitory	11 µM;	PTP1B (IC_50_);	[93]
		27 µM	TCPTP (IC_50_)	
**Lactones**				
Cladosporamide A (**75**)	Enzymatic inhibitory	48 µM;	PTP1B (IC_50_);	[93]
		54 µM	TCPTP (IC_50_)	
**Macrolides**				
Cladocladosin A (**80**)	Antimicrobial	16 µg mL^−1^;	*E. coli* (MIC);	[34]
		1 µg mL^−1^;	*E. tarda* (MIC);	
		4 µg mL^−1^;	*P. aeruginosa* (MIC);	
		2 µg mL^−1^;	*Vibrio anguillarum* (MIC);	
		32 µg mL^−1^;	*F. oxysporium f. sp. momordicae* (MIC);	
		32 µg mL^−1^;	*Penicillium digitatum* (MIC);	
		8 µg mL^−1^	*Harpophora maydis* (MIC)	
Cladospolide B (**83**)	Phytotoxic	1 µg plant^−1^	*Oryza sativa* (37.8%)	[64]
5*R*-Hydroxyrecifeiolide (**90**)	Antimicrobial	32 µg mL^−1^;	*E. ictarda* (MIC);	[32]
		32 µg mL^−1^	*P. aeruginosa* (MIC)	
5*S*-Hydroxyrecifeiolide (**91**)	Antimicrobial	16 µg mL^−1^	*G. cingulata* (MIC)	[32]
5*Z*-7-Oxozeaenol (**94**)	Phytotoxic	4.8 µg mL^−1^	*Amaranthus retroflexus* (IC_50_)	[49]
Pandangolide 1 (**95**)	Antimicrobial	32 µg mL^−1^;	*S. aureus* (MIC);	[32]
		4 µg mL^−1^;	*E. ictarda* (MIC);	
		1 µg mL^−1^;	*G. cingulata* (MIC);	
		32 µg mL^−1^	*P. aeruginosa* (MIC)	
Pandangolide 3 (**98**)	Antimicrobial	2 µg mL^−1^;	*C. gloeosporioides* (MIC);	[33]
		8 µg mL^−1^	*B. sorokiniana* (MIC)	
		32 µg mL^−1^;	*E. tarda* (MIC);	[50]
		32 µg mL^−1^;	*E. ictarda* (MIC);	
		32 µg mL^−1^;	*C. gloeosporioides* (MIC);	
		16 µg mL^−1^	*F. oxysporum* f. sp. *cucumerinum* (MIC)	
Sporiolide A (**101**)	Antimicrobial	16.7 µg mL^−1^;	*Micrococcus luteus* (MIC);	[88]
		16.7 µg mL^−1^;	*C. albicans* (MIC);	
		8.4 µg mL^−1^;	*Cryptococcus neoformans* (MIC);	
		16.7 µg mL^−1^;	*Aspergillus niger* (MIC);	
		8.4 µg mL^−1^	*Neurospora crassa* (MIC)	
	Cytotoxic	0.13 µg mL^−1^	L1210 (IC_50_)	
Sporiolide B (**102**)	Antimicrobial	16.7 µg mL^−1^	*M. luteus* (MIC)	[88]
	Cytotoxic	0.81 µg mL^−1^	L1210 (IC_50_)	
Thiocladospolide A (**103**)	Antimicrobial	1 µg mL^−1^;	*E. tarda* (MIC);	[33]
		8 µg mL^−1^;	*E. ictarda* (MIC);	
		2 µg mL^−1^	*C. glecosporioides* (MIC)	
		32 µg mL^−1^;	*E. tarda* (MIC);	[50]
		32 µg mL^−1^;	*E. ictarda* (MIC);	
		16 µg mL^−1^	*C. gloeosporioides* (MIC)	
Thiocladospolide B (**104**)	Antimicrobial	2 µg mL^−1^;	*C. gloeosporioides* (MIC);	[33]
		32 µg mL^−1^;	*Physalospora piricola* (MIC);	
		1 µg mL^−1^ù	*F. oxysporum* (MIC)	
Thiocladospolide C (**105**)	Antimicrobial	1 µg mL^−1^;	*C. gloeosporioides* (MIC);	[33]
		32 µg mL^−1^;	*P. piricola* (MIC);	
		32 µg mL^−1^	*F. oxysporum* (MIC)	
Thiocladospolide D (**106**)	Antimicrobial	1 µg mL^−1^;	*E. ictarda* (MIC);	[33]
		1 µg mL^−1^;	*C. gloeosporioides* (MIC);	
		32 µg mL^−1^;	*P. piricola* (MIC);	
		1 µg mL^−1^	*F. oxysporum* (MIC)	
Thiocladospolide F (**108**)	Antimicrobial	16 µg mL^−1^;	*E. coli* (MIC);	[34]
		2 µg mL^−1^;	*E. tarda* (MIC);	
		2 µg mL^−1^;	*V. anguillarum* (MIC);	
		16 µg mL^−1^;	*F. oxysporium f. sp. momordicae* (MIC);	
		16 µg mL^−1^;	*P. digitatum* (MIC);	
		4 µg mL^−1^	*H. maydis* (MIC)	
Thiocladospolide F *bis* (**109**)	Antimicrobial	32 µg mL^−1^;	*E. tarda* (MIC);	[50]
		16 µg mL^−1^;	*E. ictarda* (MIC);	
		16 µg mL^−1^	*B. sorokiniana* (MIC)	
Thiocladospolide G (**110**)	Antimicrobial	2 µg mL^−1^;	*E. tarda* (MIC);	[34]
		2 µg mL^−1^;	*V. anguillarum* (MIC);	
		32 µg mL^−1^;	*F. oxysporium f. sp. momordicae* (MIC);	
		32 µg mL^−1^;	*P. digitatum* (MIC);	
		8 µg mL^−1^	*H. maydis* (MIC)	
Thiocladospolide G *bis* (**111**)	Antimicrobial	4 µg mL^−1^;	*E. tarda* (MIC);	[50]
		32 µg mL^−1^;	*E. ictarda* (MIC);	
		32 µg mL^−1^;	*C. mandshurica Miura* (MIC);	
		16 µg mL^−1^;	*C. gloeosporioides* (MIC);	
		32 µg mL^−1^	*F. oxysporum* f. sp. *cucumerinum* (MIC)	
Thiocladospolide H (**112**)	Antimicrobial	16 µg mL^−1^;	*E. tarda* (MIC);	[50]
		8 µg mL^−1^;	*E. ictarda* (MIC);	
		16 µg mL^−1^;	*C. gloeosporioides* (MIC);	
		16 µg mL^−1^	*B. sorokiniana* (MIC)	
Thiocladospolide I (**113**)	Antimicrobial	32 µg mL^−1^;	*E. tarda* (MIC);	[50]
		32 µg mL^−1^;	*E. ictarda* (MIC);	
		16 µg mL^−1^	*F. oxysporum* f. sp. *cucumerinum* (MIC)	
Thiocladospolide J (**114**)	Antimicrobial	16 µg mL^−1^;	*E. tarda* (MIC);	[50]
		16 µg mL^−1^;	*E. ictarda* (MIC);	
		16 µg mL^−1^;	*C. mandshurica Miura* (MIC);	
		16 µg mL^−1^;	*C. gloeosporioides* (MIC);	
		32 µg mL^−1^;	*B. sorokiniana* (MIC);	
		16 µg mL^−1^	*F. oxysporum* f. sp. *cucumerinum* (MIC)	
Zeaenol (**115**)	Phytotoxic	8.16 µg mL^−1^	*A. retroflexus* (IC_50_)	[49]
**Naphthalene derivatives**				
Cladonaphchrom A (**116**)	Antimicrobial	1.25 µg mL^−1^;	*Scaphirhynchus albus* (MIC);	[83]
		2.5 µg mL^−1^;	*E. coli* (MIC);	
		10 µg mL^−1^;	*B. subtilis* (MIC);	
		5 µg mL^−1^;	*Micrococcus tetragenus* (MIC);	
		10 µg mL^−1^;	*M. luteus* (MIC);	
		50 µg mL^−1^;	*Alternaria brassicicola* (MIC);	
		50 µg mL^−1^;	*Phytophthora parasitica var. nicotianae* (MIC);	
		25 µg mL^−1^;	*Colletotrichum capsici* (MIC);	
		100 µg mL^−1^;	*B. oryzae* (MIC);	
		50 µg mL^−1^;	*Diaporthe medusaea* (MIC);	
		50 µg mL^−1^	*Cyanophora paradoxa* (MIC)	
Cladonaphchrom B (**117**)	Antibacterial	2.5 µg mL^−1^;	*S. albus* (MIC);	[83]
		2.5 µg mL^−1^;	*E. coli* (MIC);	
		5 µg mL^−1^;	*B. subtilis* (MIC);	
		5 µg mL^−1^;	*M. tetragenus* (MIC);	
		10 µg mL^−1^;	*M. luteus* (MIC);	
		25 µg mL^−1^;	*A. brassicicola* (MIC);	
		50 µg mL^−1^;	*P. parasitica var. nicotianae* (MIC);	
		25 µg mL^−1^;	*C. capsici* (MIC);	
		100 µg mL^−1^;	*D. medusaea* (MIC);	
		50 µg mL^−1^	*C. paradoxa* (MIC)	
**Naphtalenones**				
Altertoxin XII (**120**)	Quorum sensing inhibitory	20 µg well^−1^	*Chromobacterium violaceum* (MIC)	[87]
Cladosporol A (**121**)	Antifungal	100 ppm	*Uromyces appendiculatus* (84.2%)	[66]
	Β-1,3-glucan biosynthesis inhibitor	10 µg ml	IC_50_	[26]
Cladosporol B (**122**)	Antifungal	100 ppm	*U. appendiculatus* (100%)	[66]
Cladosporol C (**123**)	Antifungal	100 ppm	*U. appendiculatus* (77.6%)	[66]
	Antibacterial	8 µg mL^−1^;	*E. coli* (MIC);	[39]
		64 µg mL^−1^;	*M. luteus* (MIC);	
		16 µg mL^−1^	*Vibrio harveyi* (MIC)	
	Cytotoxic	33.9 µM;	*A549 (100%);*	[86]
		45.6 µM;	H1975 (100%);	
		72.5 µM;	HL60 (100%);	
		11.4 µM	MOLT-4 (100%)	
		14 µM;	A549 (IC_50_);	[39]
		4 µM	H446 (IC_50_)	
Cladosporol D (**124**)	Antifungal	100 ppm	*U. appendiculatus* (69.4%)	[66]
	Anti-COX-2	60.2 µM	IC_50_	[86]
Cladosporol E (**125**)	Antifungal	100 ppm	*U. appendiculatus* (74.8%)	[66,85]
Cladosporol F (**126**)	Antibacterial	32 µg mL^−1^;	*E. coli* (MIC);	[39]
		64 µg mL^−1^;	*M. luteus* (MIC);	
		32 µg mL^−1^	*V. harveyi* (MIC)	
	Cytotoxic	15 µM;	A549 (IC_50_);	[39,40]
		10 µM;	HeLa (IC_50_);	
		23 µM;	K562 (IC_50_);	
		23 µM	HCT-116 (IC_50_)	
Cladosporol G (**127**)	Cytotoxic	3.9 µM;	HeLa (IC_50_);	[40]
		8.8 µM;	K562 (IC_50_);	
		19.5 µM	HCT-116 (IC_50_)	
Cladosporol G *bis* (**128**)	Antibacterial	64 µg mL^−1^;	*E. coli* (MIC);	[39]
		128 µg mL^−1^;	*M. luteus* (MIC);	
		64 µg mL^−1^	*V. harveyi* (MIC)	
	Cytotoxic	13 µM;	A549 (IC_50_);	
		11 µM;	HeLa (IC_50_);	
		10 µM;	Huh7 (IC_50_);	
		11 µM;	L02 (IC_50_);	
		14 µM;	LM3 (IC_50_);	
		15 µM	SW1990 (IC_50_)	
Cladosporol H (**129**)	Antibacterial	32 µg mL^−1^;	*E. coli* (MIC);	[39]
		64 µg mL^−1^;	*M. luteus* (MIC);	
		4 µg mL^−1^	*V. harveyi* (MIC)	
	Cytotoxic	5 µM;	A549 (IC_50_);	
		10 µM;	H446 (IC_50_);	
		1 µM;	Huh7 (IC_50_);	
		4.1 µM;	LM3 (IC_50_);	
		10 µM;	MCF-7 (IC_50_);	
		14 µM	SW1990 (IC_50_)	
Cladosporol I (**130**)	Quorum sensing inhibitory	30 µg well^−1^	*C. violaceum* (MIC)	[87]
	Antibacterial	64 µg mL^−1^;	*E. coli* (MIC);	[39]
		64 µg mL^−1^;	*M. luteus* (MIC);	
		16 µg mL^−1^	*V. harveyi* (MIC)	
	Cytotoxic	10.8 µM	HeLa (IC_50_)	
Cladosporol J (**131**)	Antibacterial	16 µg mL^−1^;	*E. coli* (MIC);	[39]
		64 µg mL^−1^;	*M. luteus* (MIC);	
		32 µg mL^−1^	*V. harveyi* (MIC)	
	Cytotoxic	15 µM;	*A549 (IC_50_);*	
		4 µM;	H446 (IC_50_);	
		4.9 µM;	HeLa (IC_50_);	
		6.2 µM;	Huh7 (IC_50_);	
		13 µM;	L02 (IC_50_);	
		9.1 µM;	LM3 (IC_50_);	
		1.8 µM;	MCF-7 (IC_50_);	
		2.2 µM	SW1990 (IC_50_)	
Cladosporone A (**132**)	Cytotoxic	14.3 µM;	K562 (100%);	[86]
		15.7 µM;	A549 (100%);	
		29.9 µM;	Huh-7 (100%);	
		40.6 µM;	H1975 (100%);	
		21.3 µM;	MCF-7 (100%):	
		10.5 µM;	U937 (100%);	
		17.0 µM;	BGC823 (100%);	
		10.1 µM;	HL60 (100%);	
		53.7 µM;	HeLa (100%)	
		14.6 µM	MOLT-4 (100%)	
	Anti-COX-2	49.1 µM	IC_50_	
(3*S*)-3,8-Dihydroxy-6,7-dimethyl-α- tetralone (**137**)	Antibacterial	20 µM	*S. aureus*, *B. cereus*, *E. coli*, *Vibrio alginolyticus*, *Vibrio parahemolyticus*, methicillin-resistant *S. aureus*	[81]
Scytalone (**139**)	Antibacterial	63.6 µg mL^−1^;	*Bacillus cereus* (IC_50_);	[68]
		95.5 µg mL^−1^	*E. coli* (IC_50_)	
**Naphtoquinones and anthraquinones**				
Anhydrofusarubin (**142**)	Cytotoxic	3.97 μg mL^–1^	K-562 (IC_50_)	[90]
Methyl ether of fusarubin (**143**)	Cytotoxic	3.58 μg mL^–1^	K-562 (IC_50_)	[90]
	Antibacterial	40 μg disc^–1^	*S. aureus* (27 mm), *Bacillus megaterium* (22 mm), *E coli* (25 mm), *P. aeruginosa* (24 mm)	
	Toxic	81.4 µM	brine shrine naupalii (IC_50_)	
**Perylenequinones**				
Altertoxin VIII (**145**)	Quorum sensing inhibitory	30 µg well^−1^	*C. violaceum* (MIC)	[87]
Altertoxin IX (**146**)	Quorum sensing inhibitory	30 µg well^−1^	*C. violaceum* (MIC)	[87]
Aterotoxin X (**147**)	Quorum sensing inhibitory	20 µg well^−1^	*C. violaceum* (MIC)	[87]
Altertoxin XI (**148**)	Quorum sensing inhibitory	30 µg well^−1^	*C. violaceum* (MIC)	[87]
Calphostin A (=UCN-1028A) (**149**)	PK inhibition	0.19 µg mL^−1^;	PKC (IC_50_);	[29,30]
		40 µg mL^−1^	PKA (IC_50_)	
	Cytotoxic	0.29 µg mL^−1^;	HeLa S3 (IC_50_);	
		0.21 µg mL^−1^	MCF-7 (IC_50_)	
Calphostin B (**150**)	PK inhibition	1.04 µg mL^−1^;	PKC (IC_50_);	[7]
		22.9 µg mL^−1^	PKA (IC_50_)	
	Cytotoxic	2.56 µg mL^−1^;	HeLa S3 (IC_50_);	
		1.61 µg mL^−1^	MCF-7 (IC_50_)	
Calphostin C (=cladochrome E) (**151**)	PK inhibition	0.05 µg mL^−1^	PKC (IC_50_)	[30]
	Cytotoxic	0.23 µg mL^−1^;	HeLa S3 (IC_50_);	
		0.18 µg mL^−1^	MCF-7 (IC_50_)	
Calphostin D (= ent-isophleinchrome) (**152**)	PK inhibition	6.36 µg mL^−1^;	PKC (IC_50_);	[30]
		12.7 µg mL^−1^	PKA (IC_50_)	
	Cytotoxic	8.45 µg mL^−1^;	HeLa S3 (IC_50_);	
		2.69 µg mL^−1^	MCF-7 (IC_50_)	
	Phytotoxic	5 µg L^−1^	Sugar beet cells (100% inhibition in the light, 37–64% inhibition in the dark)	[46]
		33 µg L^−1^	Necrosis on sugar beet leaves	
Calphostin I (= Cladochrome D) (**153**)	PK inhibition	6.36 µg mL^−1^;	PKC (IC_50_);	[30]
		12.7 µg mL^−1^	PKA (IC_50_)	
	Cytotoxic	0.24 µg mL^−1^;	HeLa S3 (IC_50_);	
		0.16 µg mL^−1^	MCF-7 (IC_50_)	
Phleichrome (**154**)	Invertase I inhibition	0.5 mM	62%	[99]
**Seco acids**				
Cladospolide E (**159**)	Lipid accumulation	10 µM	Oleic acid; Triglycerides (~170 µg mg^−1^ protein); Total cholesterol (~3 µg mg^−1^ protein)	[94]
Seco-patulolide A (**161**)	Lipid accumulation	10 µM	Oleic acid; Triglycerides (~150 µg mg^−1^ protein); Total cholesterol (~3 µg mg^−1^ protein)	[94]
Seco-patulolide C (**162**)	Lipid accumulation	10 µM	Oleic acid; Triglycerides (~150 µg mg^−1^ protein); Total cholesterol (~3 µg mg^−1^ protein)	[94]
	Antimicrobial	32 µg mL^−1^;	*E. tarda* (MIC);	[50]
		32 µg mL^−1^;	*E. ictarda* (MIC);	
		16 µg mL^−1^	*C. gloeosporioides* (MIC)	
(3*S*,5*S*,11*S*)-Trihydroxydodecanoic acid (**163**)	Antibacterial	63.6 µg mL^−1^	*B. cereus* (MIC)	[68]
	Cytotoxic	42 µM;	MCF-7;	
		82 µM	T47D	
	Antibacterial	40 μg disc^–1^	*S. aureus* (27 mm), *B. megaterium* (22 mm), *E coli* (25 mm), *P. aeruginosa* (24 mm)	
	Toxic	81.4 µM	brine shrine naupalii (IC_50_)	
**Sterols**				
Cladosporide A (**164**)	Antifungal	0.5 µg mL^−1^	*Aspergillus fumigatus* (IC_50_)	[79]
Cladosporide B (**165**)	Antifungal	3 µg disc^−1^	*A. fumigatus* (11 mm)	[80]
Cladosporide C (**166**)	Antifungal	1.5 µg disc^−1^	*A. fumigatus* (11 mm)	[80]
3α-Hydroxy-pregn-7-ene-6,20-dione (**171**)	Anti-adipogenic	1.25 – 10 µM	3T3-L1	[62]
**Tetramic acids**				
Cladodionen (**176**)	Cytotoxic	28.6 µM	HL-60 (IC_50_)	[58]
		4.5 µM;	K562 (IC_50_);	[89]
		6.6 µM;	HL-60 (IC_50_);	
		12 µM;	HCT-116 (IC_50_);	
		11 µM;	PC-3 (IC_50_);	
		15 µM;	SH-SYSY (IC_50_);	
		22 µM	MGC-803 (IC_50_)	
		18.7 µM;	MCF-7 (IC_50_);	[96]
		19.1 µM;	HeLa (IC_50_);	
		17.9 µM;	HCT-116 (IC_50_);	
		9.1 µM	HL-60 (IC_50_)	
		3.7 µM	L5178 (IC_50_)	[63]
	Antifungal	100 mg/plate;	*Ustilago maydis* (0.97 cm);	
		100 mg/plate	*Saccharomyces cerevisiae* (3.27 cm)	
Cladosin B (**178**)	Renoprotective effects against cisplatin-induced kidney cell damage	25 µM; 50 µM; 100 µM	LLC-PK1 (dose-dependent)	[60]
Cladosin C (**179**)	Antiviral	276 µM	H1N1 (IC_50_)	[55]
Cladosin F (**181**)	Renoprotective effects against cisplatin-induced kidney cell damage	25 µM; 50 µM; 100 µM	LLC-PK1 (dose-dependent)	[60]
Cladosin I (**184**)	Cytotoxic	4.1 µM;	K562 (IC_50_);	[89]
		2.8 µM;	HL-60 (IC_50_);	
		11 µM;	HCT-116 (IC_50_);	
		13 µM;	PC-3 (IC_50_);	
		12 µM;	SH-SYSY (IC_50_);	
		19 µM	MGC-803 (IC_50_)	
Cladosin J (**185**)	Cytotoxic	6.8 µM;	K562 (IC_50_);	[89]
		7.8 µM	HL-60 (IC_50_)	
Cladosin K (**186**)	Cytotoxic	5.9 µM;	K562 (IC_50_);	[89]
		7.5 µM;	HL-60 (IC_50_);	
		14 µM;	HCT-116 (IC_50_);	
		18 µM	PC-3 (IC_50_)	
Cladosin L (**187**)	Renoprotective effects against cisplatin-induced kidney cell damage	25 µM; 50 µM; 100 µM	LLC-PK1 (dose-indipendent)	[60]
Cladosin L *bis* (**188**)	Antibacterial	25 µM;	*S. aureus* ATCC 700699 (IC_50_);	[63]
		50 µM	*S. aureus* ATCC 29213 (IC_50_)	
Cladosporicin A (**192**)	Cytotoxic	70.88 µM;	Bt549 (IC_50_);	[61]
		74.48 µM;	HCC70 (IC_50_);	
		75.54 µM;	MDA-MB-231 (IC_50_);	
		79.36 µM	MDA-MB-468 (IC_50_)	
Cladosporiumin I *bis* (**202**)	Cytotoxic	76.18 µM;	Bt549 (IC_50_);	[61]
		85.29 µM;	HCC70 (IC_50_);	
		82.37 µM;	MDA-MB-231 (IC_50_);	
		81.44 µM	MDA-MB-468 (IC_50_)	
Cladosporiumin J *bis* (**204**)	Cytotoxic	78.96 µM;	Bt549 (IC_50_);	[61]
		76.41 µM;	HCC70 (IC_50_);	
		79.27 µM;	MDA-MB-231 (IC_50_);	
		74.64 µM	MDA-MB-468 (IC_50_)	
**Tropolones**				
Malettinin A (**210**)	Antimicrobial	33.1 µM;	*Trichophyton rubrum* (IC_50_);	[95]
		100 µM	*C. albicans* (81%)	
Malettinin B (**211**)	Antimicrobial	28.3 µM;	*Xanthomonas campestris* (IC_50_);	[95]
		60.6 µM;	*T. rubrum* (IC_50_);	
		100 µM;	*Staphylococcus epidermidis* (<80%);	
		100 µM;	*B. subtilis* (<80%);	
		100 µM	*C. albicans* (<80%)	
Malettinin C (**212**)	Antimicrobial	37.9 µM;	*X. campestris* (IC_50_);	[95]
		83.2 µM;	*T. rubrum* (IC_50_);	
		100 µM;	*S. epidermidis* (<80%);	
		100 µM;	*B. subtilis* (<80%);	
		100 µM	*C. albicans* (<80%)	
Malettinin E (**213**)	Antimicrobial	28.7 µM;	*X. campestris* (IC_50_);	[95]
		30.7 µM;	*T. rubrum* (IC_50_)	
**Xanthones**				
Conioxanthone A (**218**)	Cytotoxic	10 µM	22RV1 (36.8%), C4-2B (3.3%), RWPE-1 (20.3%)	[43]
3,8-Dihydroxy-6-methyl-9-oxo-9*H*-xanthene-1-carboxylate (**219**)	Cytotoxic	10 µM	22RV1 (82.1%), C4-2B (77.7%), RWPE-1 (11.5%)	[43]
α-Diversonolic ester (**220**)	Cytotoxic	10 µM	22RV1 (28.8%), C4-2B (12.9%), RWPE-1 (24.3%)	[43]
β-Diversonolic ester (**221**)	Cytotoxic	10 µM	22RV1 (40.2%), C4-2B (2.8%), RWPE-1 (10.3%)	[43]
8-Hydroxy-6-methylxanthone-1-carboxylic acid (**222**)	Cytotoxic	10 µM	22RV1 (71.3%), C4-2B (60.7%), RWPE-1 (19.7%)	[43]
Methyl 8-hydroxy-6-(hydroxymethyl)- 9-oxo-9*H*-xanthene-1-carboxylate (**223**)	Cytotoxic	10 µM	22RV1 (68.1%), C4-2B (20.2%), RWPE-1 (19.0%)	[43]
Methyl 8-hydroxy-6-methyl-9-oxo-9*H*-xanthene-1-carboxylate (**224**)	Cytotoxic	10 µM	22RV1 (55.8%), C4-2B (8.1%), RWPE-1 (5.3%)	[43]
8-(Methoxycarbonyl)-1-hydroxy-9-oxo-9*H*-xanthene-3-carboxylic acid (**225**)	Cytotoxic	10 µM	22RV1 (63.9%), C4-2B (12.2%), RWPE-1 (27.0%)	[43]
Vertixanthone (**227**)	Cytotoxic	10 µM	22RV1 (27.1%), RWPE-1 (25.0%)	[43]
**Miscellaneous**				
Acetyl Sumiki’s acid (**229**)	Antibacterial	5 μg disc^−1^	*B. subtilis* (7 mm), *S. aureus* (7 mm)	[44]
1,1′-Dioxine-2,2′-dipropionic acid (**233**)	Antibacterial	25 μg mL^−1^;	*S. aureus* (MIC);	[85]
		25 μg mL^−1^;	*E. coli* (MIC);	
		12.5 μg mL^−1^	*B. cereus* (MIC)	
4-*O*-α-*d*-Ribofuranose-2-pentyl-3-phemethylol (**238**)	Inhibition of α-glucosidase	2.50 µM	IC_50_	[82]
Sumiki’s acid (**241**)	Antibacterial	5 μg disc^−1^	*B. subtilis* (7 mm), *S. aureus* (7 mm)	[44]
Taxol (**242**)	Cytotoxic	3.5 µM	HCT 15 (IC_50_)	[51]
	Antibacterial	30 µL disc^−1^;	*Pseudomonas aeruginosa* (2 mm);	
		20 µL disc^−1^;	*Escherichia coli* (3 mm);	
		30 µL disc^−1^;	*Klebsiella pneumoniae* (2 mm);	
		20 µL disc^−1^;	*Acetobacter* sp. (2 mm);	
		40 µL disc^−1^	*Bacillus subtilis* (1 mm)	
Vermistatin (**244**)	Antibacterial	25 μg mL^−1^;	*S. aureus* (MIC);	[85]
		25 μg mL^−1^	*B. cereus* (MIC)	

## Data Availability

Not applicable.
